# Lineage specific evolution of the VNTR composite retrotransposon central domain and its role in retrotransposition of gibbon LAVA elements

**DOI:** 10.1186/s12864-015-1543-z

**Published:** 2015-05-16

**Authors:** Iulia Lupan, Paul Bulzu, Octavian Popescu, Annette Damert

**Affiliations:** Institute for Interdisciplinary Research in Bio-Nano-Sciences, Molecular Biology Center, Babes-Bolyai-University, Treboniu Laurian Street 42, Cluj-Napoca, RO-400271 Romania; Institute of Biology, Romanian Academy, Bucharest, Romania

**Keywords:** SVA, Retrotransposon, Tandem repeats, Hominoids

## Abstract

**Background:**

VNTR (Variable Number of Tandem Repeats) composite retrotransposons - SVA (SINE-R-VNTR-*Alu*), LAVA (LINE-1-*Alu*-VNTR-*Alu*), PVA (*PTGR2*-VNTR-*Alu*) and FVA (FRAM-VNTR-*Alu*) - are specific to hominoid primates. Their assembly, the evolution of their 5’ and 3’ domains, and the functional significance of the shared 5’ *Alu*-like region are well understood. The central VNTR domain, by contrast, has long been assumed to represent a more or less random collection of 30-50 bp GC-rich repeats. It is only recently that it attracted attention in the context of regulation of SVA expression.

**Results:**

Here we provide evidence that the organization of the VNTR is non-random, with conserved repeat unit (RU) arrays at both the 5’ and 3’ ends of the VNTRs of human, chimpanzee and orangutan SVA and gibbon LAVA. The younger SVA subfamilies harbour highly organized internal RU arrays. The composition of these arrays is specific to the human/chimpanzee and orangutan lineages, respectively. Tracing the development of the VNTR through evolution we show for the first time how tandem repeats evolve within the constraints set by a functional, non-autonomous non-LTR retrotransposon in two different families - LAVA and SVA - in different hominoid lineages. Our analysis revealed that a microhomology-driven mechanism mediates expansion/contraction of the VNTR domain at the DNA level.

Elements of all four VNTR composite families have been shown to be mobilized by the autonomous LINE1 retrotransposon in *trans*. In case of SVA, key determinants of mobilization are found in the 5’ hexameric repeat/*Alu*-like region. We now demonstrate that in LAVA, by contrast, the VNTR domain determines mobilization efficiency in the context of domain swaps between active and inactive elements.

**Conclusions:**

The central domain of VNTR composites evolves in a lineage-specific manner which gives rise to distinct structures in gibbon LAVA, orangutan SVA, and human/chimpanzee SVA. The differences observed between the families and lineages are likely to have an influence on the expression and mobilization of the elements.

**Electronic supplementary material:**

The online version of this article (doi:10.1186/s12864-015-1543-z) contains supplementary material, which is available to authorized users.

## Background

**Figure 1:**
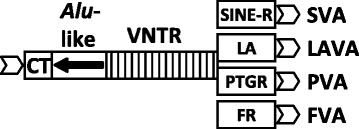
VNTR composite retrotransposon families in hominoid primates. All families share the 5’ CT repeat/*Alu*-like region and the central VNTR, but are characterized by variant 3’ ends. In SVA the 3’ part is composed of a sequence derived from the human endogenous retrovirus HERV-K (SINE-R). The 3’ part of LAVA elements (LA) is constituted of fragments of an *Alu*Sz (A) and of an L1ME (L) element combined with unique (U) sequences preceding the *Alu* moiety and separating it from the L1ME fragment. It is also referred to as U1-*Alu*Sz-U2-L1ME [2]. PVA elements are characterized by fusion of *PTGR2* exon 4 and part of intron 4 to the VNTR. The FVA 3’ end contains part of a FRAM (Free Right *Alu* Monomer) element surrounded by non-repetitive sequence. Chevron arrows indicate target site duplications.

VNTR (Variable Number of Tandem Repeats) composites are non-autonomous, non-LTR retrotransposons specific to hominoid primates. The group comprises SVA (SINE-R-VNTR-*Alu*) [1], LAVA (LINE-1-*Alu*-VNTR-*Alu*) [2], PVA (*PTGR2*-VNTR-*Alu*) [3] and FVA (FRAM-VNTR-*Alu*) (SINE-R - SINE of retroviral origin, *PTGR2* - *prostaglandin reductase 2*, FRAM - Free Right *Alu* Monomer) [4]. Whereas SVA elements are found in all hominoids [5], LAVA, PVA and FVA are restricted to gibbons [2-4]. All four families share the 5’ CT-hexameric repeat/*Alu*-like region and the central VNTR domain (Figure 1). SVA [6,7], as well as LAVA, PVA and FVA [4] are mobilized by the autonomous non-LTR retrotransposon LINE-1 (L1) in *trans*. The hexameric repeat/*Alu*-like 5‘ region constitutes the minimal active human SVA [8]. The sequence and derived structure of this domain has also been shown to influence mobilization efficiency of PVA and FVA [4]. By definition the 5’ hexameric repeat region of SVAs and related non-LTR retrotransposons is a VNTR. To clearly distinguish it from the central domain of the elements we will, however, refer to it as CT- or hexameric repeats throughout this article.

The central VNTR domain of SVAs is comprised of 30 to 50 bp GC-rich repeats [9]. The VNTR of the evolutionary younger human SVA subfamilies has been shown to be composed of two distinct parts (termed TR – Tandem Repeat and VNTR) [10]. Complete deletion of the VNTR in the context of a human SVA resulted in a significant reduction in *trans* mobilization rates, whereas partial deletion led to an increase in retrotransposition [8]. Recently, the SVA VNTR has been identified as the “prime interaction site of ZNF91” – a zinc finger protein that mediates transcriptional repression [11].

Involvement in transcriptional regulation has been demonstrated for a number of different VNTRs in the human genome. Activity of the *monoamine oxidase A* promoter, for example, is affected by two VNTRs in a repeat number dependent manner [12,13]. Similar effects have been reported for the serotonin transporter gene VNTRs located upstream of the gene [14] and in the second intron [15], and a VNTR in the promoter of the thiopurine methyltransferase gene [16]. Polymorphism in the VNTR found in the 3’UTR of the dopamine transporter gene appears to influence gene expression at the post-transcriptional level [17].

In the process of cloning LAVA and SVA elements for functional studies we noticed that (i) the LAVA_E element found to be inactive in our *trans*-mobilization assays displayed a particular repeat unit structure at the VNTR 5’ end, which was not shared by any of the other VNTR composites cloned and analyzed, and (ii) in contrast to SVA the LAVA VNTRs represented nearly perfect EagI repeats. These findings could possibly serve to explain the inactivity of the LAVA_E element and the incompatibility of SVA and LAVA in the context of chimeras [4], respectively. Therefore, we analyzed a small sample set to investigate whether the above observations were valid across a larger number of elements. As this was found to be the case, we initiated a more comprehensive and detailed analysis across all families/subfamilies of VNTR composites in all hominoids except gorilla.

Here we report that VNTR repeat units have evolved over time, creating subsets specific for particular subfamilies of SVA and LAVA. We show that the VNTRs of the evolutionary younger human and chimpanzee SVA subfamilies are composed of highly organized repeat unit arrays. A similar tendency can be observed in the younger orangutan SVA subfamilies. In LAVA at most five repeat units are conserved at the 5’ and 3’ ends of the domain, respectively. Comparison of orthologous SVA_D elements in human and chimpanzee reveals a microhomology-driven mechanism mediating VNTR remodelling at the DNA level. Finally, we provide evidence that key determinants of LAVA mobilization are localized in the VNTR region.

## Results

The datasets used in the analysis are summarized in Table 1.

**Table 1:** **Datasets used in the study**

**Species**	**VNTR composite family**	**Subfamilies**	**Number of elements analyzed**	**Remarks**
*Macaca mulatta* (MMU)	SVA2_MMU_		30	
*Nomascus leucogenys* (NLE)	SVA2_NLE_		30	
	SVA_NLE_ (SINE-R-VNTR-*Alu*)		26	
	PVA (*PTGR2*-VNTR-*Alu*)		89	Only elements displaying a 5’ complete VNTR (i.e. containing at least the 3’ part of the *Alu*-like region)
	FVA (FRAM-VNTR-*Alu*)		7	
	LAVA (L1-*Alu*-VNTR-*Alu*)	LAVA_A – LAVA_F (22 subfamilies)	5, 10 or 20 per subfamily, representing 5-10% of the subfamily members	Only elements displaying a 5’ complete VNTR (i.e. containing at least the 3’ part of the *Alu*-like region)
*Pongo abelii* (PA)	SVA (SINE-R-VNTR-*Alu*)	SVA_PA__1 - SVA_PA__11 (11 subfamilies)	5, 10 or 20 per subfamily	For the younger subfamilies SVA_PA__7 to SVA_PA__11 5’ truncated elements and elements with assembly gaps had to be taken into account to obtain consensus VNTR schemes with sufficient support
*Pan troglodytes* (Pt)	SVA (SINE-R-VNTR-*Alu*)	SVA_PtA	83	Only elements with full-length VNTR
*Homo sapiens* (HSA)	SVA (SINE-R-VNTR-*Alu*)	SVA_A – SVA_F (6 subfamilies)	10 per subfamily	Only full-length elements

### VNTR repeat units – variety and evolution

To establish the nature of the ancestral set of repeat units (RUs), we investigated the repeat unit composition of SVA2 elements [18-20] in *Macaca mulatta* and *Nomascus leucogenys*. SVA2 elements are the common ancestor of all VNTR composites.

**Figure 2:**
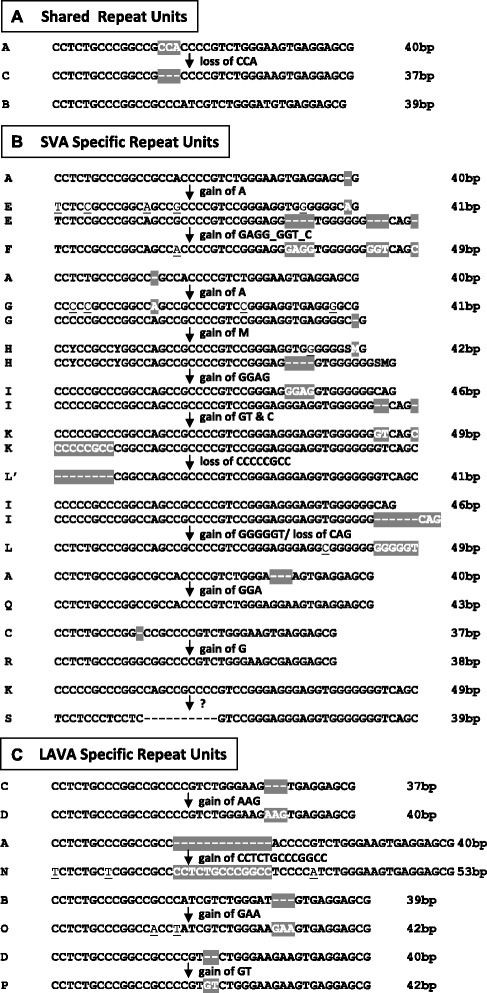
VNTR repeat unit (RU) types found in SVA and LAVA, their derivation from the basic RU types and from each other. **(A)** RU types shared between SVA and LAVA. **(B)** SVA specific RU types. **(C)** LAVA specific RU types. Indels are highlighted in grey. Nucleotides differing between consensus sequences of RU types are non-bold and underlined.

**Figure 3:**
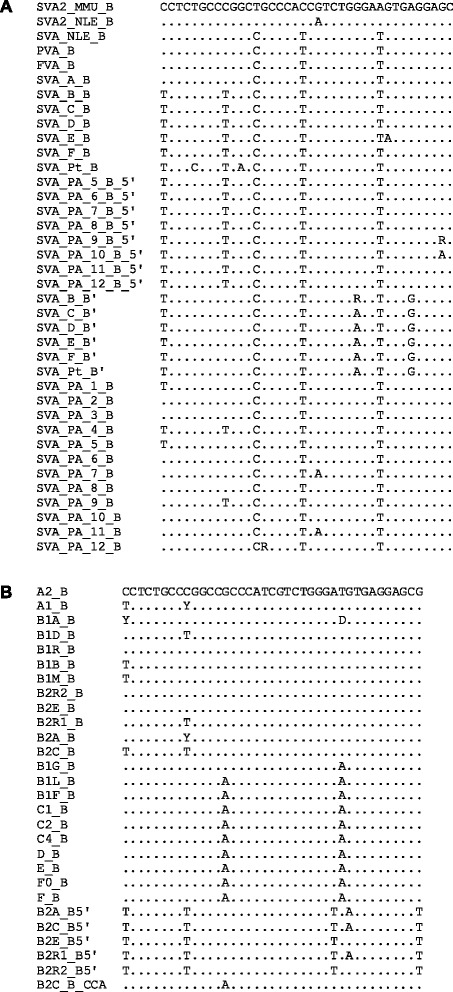
Sequence evolution in B-type repeat units. Multiple alignment of the consensus sequences of B-type repeat units of **(A)** SVA2, SVA_NLE_, PVA, FVA, SVA and **(B)** LAVA elements. B’ denotes the second B-type repeat in the 5’ repeat unit arrays of SVA_B to SVA_F. B5’ refers to the 5’-most B-type repeat unit in orangutan SVAs (in A) and LAVA_B2 (in B). B_CCA is the subfamily specific internal B-type repeat unit of LAVA_B2C. MMU – *Macaca mulatta*; NLE – *Nomascus leucogenys*; SVA_PA – orangutan (*Pongo abelii*) SVAs; SVA_PtA – chimpanzee specific SVA subfamily. The corresponding alignments for all other repeat unit types are provided in Additional file 1: Figure S1.

The analysis of 30 elements retrieved for each of the species revealed that there are two dominant repeat units of 40 and 39 bp, respectively. In view of creating an “RU code” for VNTR composites we designated these ancestral RUs A (40 bp) and B (39 bp), respectively. In addition to A and B, a number of longer as well as shorter RUs were found in SVA2 elements. None of them, however, could be identified in more than one or two elements. The subsequent analysis of the VNTR regions of LAVA and SVA subfamilies as well as of PVA and FVA identified an additional 17 repeat unit types. These were encoded C to S. Figure 2 illustrates the derivation of these from the basic RU types A and B as well as from each other. For RU type S, which is found in chimpanzee VNTRs only, it cannot be determined precisely from which other RU it has been derived. Sequence evolution is evident within some of the RU types across VNTR composite families/subfamilies. Alignments for B-type RUs are shown in Figure 3. The remaining alignments for human SVA and gibbon LAVA can be found in Additional file 1: Figure S1. Sequence variants of RUs are denoted with either prime (‘) or superscripts. In case of the younger SVA subfamilies in humans, chimpanzees and orangutans the sequence of the RUs found at particular positions is conserved (colour-coded in Additional file 2). The position – specific consensus sequences for these RUs are provided in Additional file 1: Figure S2.

### The VNTRs of human and chimpanzee SVAs are highly organized arrays of repeat units

**Figure 4:**
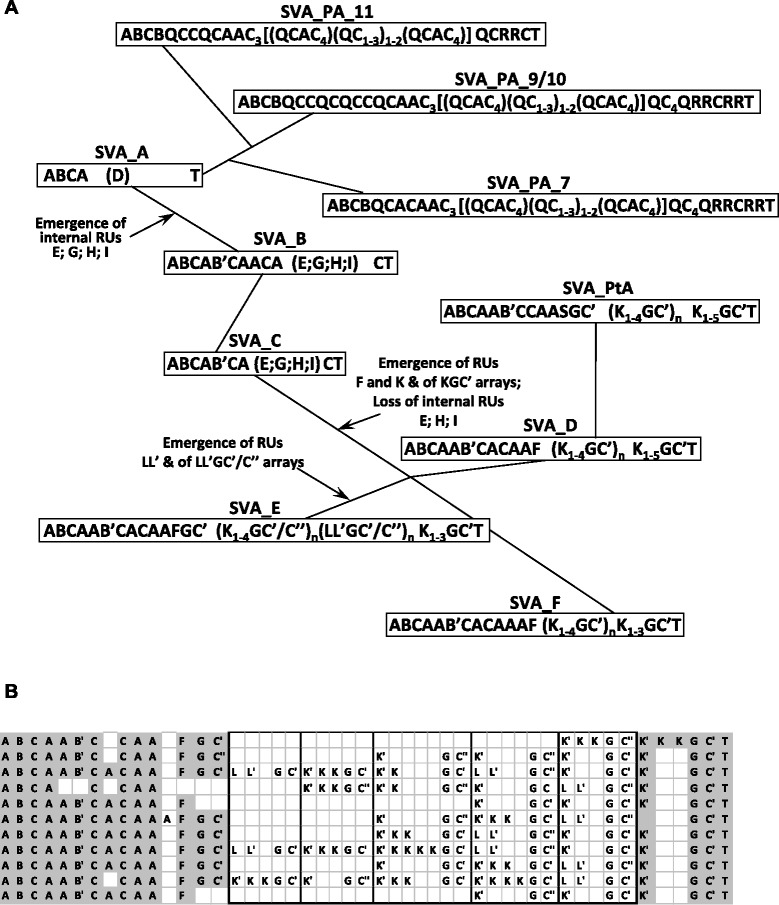
Evolution of repeat unit arrays in human, chimpanzee and orangutan SVA subfamilies. **(A)** The consensus repeat unit (RU) arrays for each of the subfamilies are superimposed on a network based on alignment of the consensus sequences of the subfamilies’ SINE-R domains ([5], details as well as a complete network for orangutan SVAs are provided in Additional file 1: Figure S3). Network branches are not drawn to scale with respect to the evolutionary distance. For reasons of clarity only the evolutionary youngest orangutan subfamilies are shown. **(B)** Repeat unit schemata for the ten SVA_E elements analyzed. Conserved arrays at the 5’ and 3’ ends are highlighted in grey; internal arrays are boxed. K’ denotes a K-type RU with a slightly divergent sequence that is always found at the 5’ end of the arrays.

**Figure 5:**
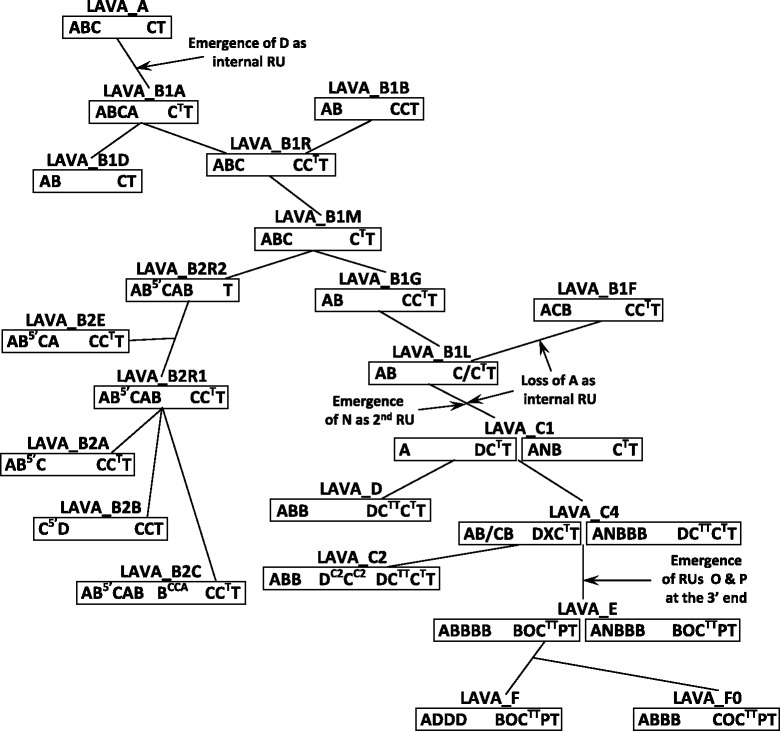
Evolution of repeat unit arrays in *Nomascus* LAVA elements. The consensus repeat unit (RU) arrays for each of the subfamilies are superimposed on a network based on alignment of the consensus sequences of the subfamilies’ 3’ U1-*Alu*Sz-U2-L1ME part (Additional file 1: Figure S4). As there are no differences to be observed between the VNTRs of LAVA_A1 and LAVA_A2, these two are presented as a single subfamily. The length of the network branches does not represent the evolutionary distance between the subfamilies. Note that in subfamilies LAVA_C1, LAVA_C4 and LAVA_E there are two different types of VNTRs.

Following the identification of the basic repeat units we next wanted to know whether they are distributed randomly over the entire length of the VNTR or whether there are specific patterns to be observed. Analysis of ten elements each derived from the human SVA subfamilies A to F revealed that the arrangement of the VNTR subunits is non-random (Figure 4, Additional file 2). A specific pattern at the 5’ end is evident in all six subfamilies. Notably, the 5’ end is always formed by an A-type repeat unit. This, apart from a small LAVA subfamily (LAVA_B2B, Figure 5), holds true for all VNTR composites (Additional file 2, Additional file 3, Additional file 4). Figure 4A shows the RU arrays found in the six human SVA subfamilies along a network generated using the SVA SINE-R part [5]. The 5’ ABCA array is found in elements of all six subfamilies. Interestingly, D-type repeat units – otherwise specific to LAVA VNTRs (see below) – are occasionally found in SVA_A elements (Additional file 2). The transition of SVA_A to SVA_B is characterized by the emergence of the longer RUs E (41 bp), G (41 bp), H (42 bp) and I (47 bp). RUs E, H and I are no longer found from SVA_D onwards (Figure 4A). Based on sequence analysis (Figure 2) we assume that they represent intermediates giving rise to the F, K and L repeat units characteristic for the younger subfamilies SVA_D, SVA_E and SVA_F. At the 5’ end the recognizable subfamily specific array expands from ABCA in SVA_A to ABCAAAB’CACAAF in SVA_F. At the VNTR 3’ end the terminal repeat unit T is preceded by a C-type repeat in SVA_B and SVA_C. The younger subfamilies SVA_D to SVA_F display the array KGC’T. Whereas there is no clearly defined interior VNTR structure to be observed in SVA_A to SVA_C, the central part of SVA_D to SVA_F VNTRs is composed exclusively of well-defined K_n_GC’ arrays. The same arrays are found in the SVA_D-derived chimpanzee specific SVA_PtA subfamily. In SVA_E LL’GC’/C” arrays are found in addition (Figure 4B).

### Lineage specific evolution of orangutan SVA VNTRs is governed by a principle similar to that in the human/chimpanzee lineage

Up to date, orangutan SVAs have been categorized as belonging to subfamilies SVA_A and SVA_B. QPCR analysis yielded an estimate of less than 1000 SVA elements in the orangutan genome [5]. Sequence analysis of the orangutan genome identified around “1800 new insertions” [21] – indicating lineage-specific amplification. Recently it has been hypothesized that the acquisition of additional Zinc fingers by the transcriptional repressor ZNF91 may have driven the evolution of new and different SVA subfamilies in the gorilla/chimpanzee/human lineage. In orangutan, “which diverged before ZNF91 had undergone these structural changes” such a pattern (i.e. the emergence of new SVA subtypes) is not observed [11]. We now provide evidence that new SVA subtypes did develop in the orangutan lineage. Analysis of the SINE-R moiety of 1128 orangutan SVA elements (excluding elements 5’ truncated in the SINE-R and those with assembly gaps in this domain) readily identified 12 subfamilies. Only 42 of the elements could be categorized as SVA_A. SVA_B elements are absent from the orangutan genome. The consensus sequences of the orangutan SVA subfamilies and a network illustrating their phylogenetic relationships are provided in Additional file 1: Figure S3. Analysis of the repeat unit content of the orangutan elements identified two orangutan specific RU types (Figure 2, Figure 4A): the 43 bp Q-type RU that has developed concomitantly with major sequence changes in the SINE-R in the transition from SVA_PA__1 (SVA_A) to SVA_PA__2 and the 38 bp R-type RU specific to the younger subfamilies SVA_PA__7-11 (Additional file 1: Figure S3). Unfortunately, the analysis of the VNTR domain of orangutan SVAs is severely hampered by assembly gaps. Especially in the younger subfamilies the complete VNTR structure could be determined only for small numbers of elements. Nevertheless, our analysis revealed a pattern of VNTR evolution that appears to be guided by the same basic principles as in the gorilla/chimpanzee/human lineage: recognizable extended 5’ RU arrays are followed by repeated arrays in the central part of the VNTR domain in the younger subfamilies. By contrast to human SVA_D to SVA_F, the subfamily-specific VNTR 3’ arrays are not identical to the expanding internal arrays in orangutan SVAs. Another notable difference to human SVAs are the RU types present in the central expanding arrays: whereas in SVA_D to SVA_F these arrays are dominated by the 49 bp G-rich K-type RUs, orangutan central arrays are characterized by amplification of C-type RUs “scaffolded” by Q-type RUs (Figure 4A, Additional file 2).

### LAVA VNTRs display conserved RU arrays only at their 5’ and 3’ ends

By comparison to SVA, LAVA elements are characterized by a smaller set of RU types. In addition to A-, B- and C-type RUs that are found in SVA as well, a second 40 bp RU, D (Figure 2C), is present in almost all LAVA subfamilies. As in all other VNTR composites the 5’ RU is always A-type, with the exception of LAVA_B2B (Figure 5). It is worthwhile noticing that in LAVA VNTRs the sequence of the 5’ A-type RU differs from that of internal A-type RUs (Additional file 1: Figure S1 B). Specific sequence variants of the RU types are found in a number of subfamilies (B^5’^ in LAVA_B2; B^CCA^ in LAVA_B2C; D^C2^ and C^C2^ in LAVA_C2; Figure 5, Additional file 3, Additional file 1: Figure S1 B).

Evolutionary analysis of LAVA VNTRs was carried out based on a network of subfamilies obtained through manual sorting of their 3’ end (U1-*Alu*Sz-U2-L1ME) sequences (Additional file 1: Figure S4). On the main network path from LAVA_A to LAVA_F (excluding the LAVA_B2 branch) six major “events” are to be observed (Figure 5): (1) the emergence of the D-type RU in the transition from LAVA_A to B1A; (2) the emergence of a slightly variant C-type RU (C^T^) as sub-terminal RU starting with B1A; (3) the loss of A as internal repeat unit on the paths leading from B1L to B1F and from B1L to C1, respectively (in B1L VNTRs both with and without A as internal RU are found); (4) the emergence of N as second RU at the VNTR 5’ end in the transition from B1L to C1; (5) the emergence of a second sub-terminal C-type repeat variant (C^TT^) on the paths leading from C1 to C4 and D, respectively, and (6) the emergence of RU types O and P as part of the terminal RU array on the path from C4 to E with concomitant loss of the C^T^ sub-terminal RU variant.

The B2 branch of the LAVA network is characterized by a shared slightly divergent B-type RU (B5’) at the second position. Two of the smaller, well-defined subfamilies (B2B and B2C) can be distinguished based on the presence of a C-type RU at the 5’ end (C^5’^ in LAVA_B2B) and a subfamily specific internal B-type RU (B^CCA^, B2C), respectively.

Overall, and in contrast to human SVAs, at most five RUs appear to be conserved across elements of the same subfamily at both the 5’ and 3’ ends. Specific patterns of organization of the VNTR internal part could not be observed – not even in the evolutionary youngest subfamilies LAVA_E and LAVA_F.

Interestingly, in subfamilies C1, C4 and E elements displaying different structures at the VNTR 5’ end are found (Figure 5). Whereas in C1 the two variants – either possessing or lacking the N-type 53 bp repeat unit at the second position – are represented equally among the elements amenable to analysis, the variant lacking the N-type RU is under-represented in C4 and E (Additional file 3). However, both these subfamilies gave rise to others (C2 in case of C4 and F in case of E) lacking the N-type RU. The source elements for further evolution towards C2 and F, respectively, can therefore be assumed to have been present in the minority fraction of their parental subfamilies.

No organized repeat unit arrays could be identified in SVA_NLE_, PVA and FVA (Additional file 4). Based on the low retrotransposition rates obtained for elements of these families *in vitro* and high divergence of their 5’ and 3’ domain sequences from the family consensus [4], we assume that they are no longer active. Most likely their VNTR regions have degenerated to an extent where an internal structure that might have existed at the time of their amplification is no longer recognizable.

### VNTR remodelling occurs at the DNA level in both SVA and LAVA

**Figure 6:**
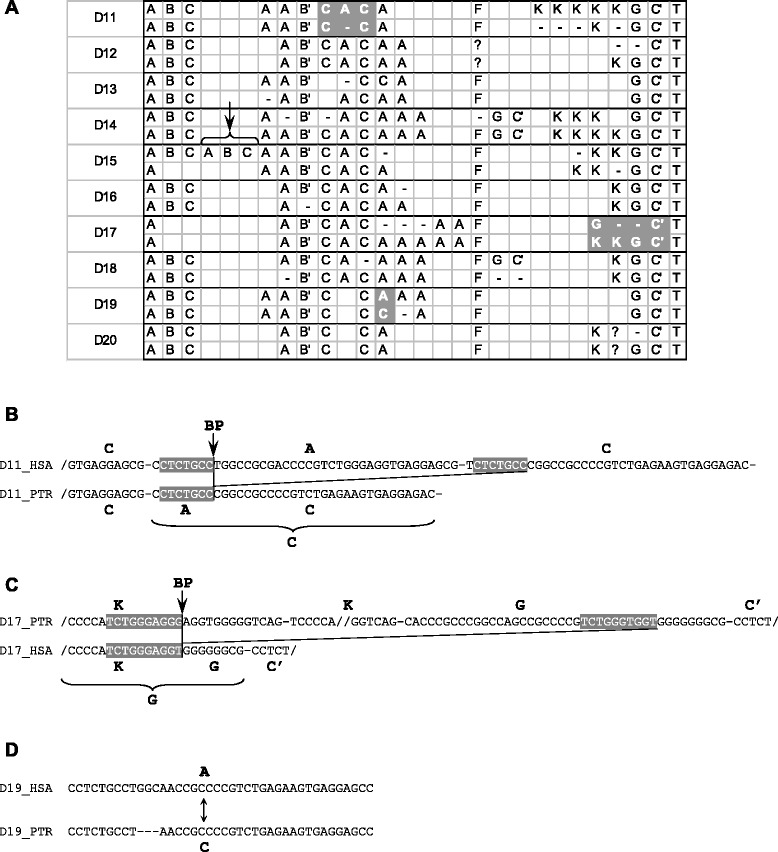
VNTR remodeling at the DNA level. **(A)** Comparison of the repeat unit schemata of orthologous SVA_D elements in human and chimpanzee. D11 to D20 are generic identifiers. The genomic positions of the elements are provided in Additional file 1. Repeat unit arrays detailed in B, C and D are highlighted in grey. A likely duplication in the human D15 ortholog is marked with an arrow. HSA – *Homo sapiens*; PTR – *Pan troglodytes*. **(B)** and **(C)** represent examples of indels resulting from a microhomology-driven mechanism. Repeat units are separated by dashes; microhomologies are highlighted in grey. Note that in (C) identity within the microhomology is not 100% - likely due to divergent sequence evolution in the two species. BP – breakpoint **(D)** shows an example of repeat unit conversion resulting from small scale insertion/deletion. The complete pairwise alignments for all ten elements analyzed are provided in Additional file 1: Figure S5.

The existence of SVA_D elements shared between human and chimpanzee provided us with the opportunity to investigate evolution of VNTRs by pairwise comparison between elements derived from a common ancestor that has integrated before the human/chimpanzee split. Overall, the orthologous elements in human and chimpanzee were found to display VNTRs more similar to each other than VNTRs of unrelated human SVA_D copies (compare Additional file 2 and Figure 6A). The formal VNTR code of the respective orthologs (Figure 6A) suggests that entire RUs have been lost or acquired. Closer inspection of the pairwise alignments (Additional file 1: Figure S5), however, shows that segments of RUs have been deleted/inserted. If deletion is assumed, then a new repeat unit is formed by combination of parts of the RUs flanking the breakpoint at its 5’ and 3’ ends (Figure 6B). In case of insertion, the newly inserted sequence would be composed of segments of different RUs. All RU copy number variations of this type (i.e. insertion/deletion across the boundaries of RUs) are characterized by microhomologies (5-42 bp) at the breakpoints (Figures 6B and C; Additional file 1: Figure S5). This suggests that a microhomology-driven mechanism mediates VNTR remodelling at the DNA level.

Only in two cases (D19, D20 – Additional file 1: Figure S5) there is precise excision/insertion of an RU. We also observed two instances of “micro” indels of 3 bp each, resulting in conversion of an A-type RU to a C-type RU or vice versa (Figure 6D; D13, D19 – Additional file 1: Figure S5).

Because of lack of information on the VNTR structure of the original elements in the human/chimpanzee ancestor it cannot be decided whether the differences in the RU patterns have resulted from insertions or deletions. However, a comparison of element D15 to the conserved RU patterns of the other elements (Figure 6A) suggests that at the VNTR 5’ end a duplication of the ABC RU array has taken place in humans.

For LAVA there is no sequence information available for elements shared between species/genera. However, a small group of LAVA elements amplified as part of segmental duplications in *Nomascus leucogenys*. Similarly to the SVA_D orthologs in human and chimpanzee, these elements are derived from a common ancestor without an RNA intermediate. Any changes in the VNTR must, therefore, have occurred at the DNA level. Alignment of the eight elements of the group reveals microhomologies at the breakpoints of all three cases of RU copy number variation (Additional file 1: Figure S6; position data of the elements are provided in Additional file 3). Thus, we conclude that the same microhomology-driven mechanism mediates VNTR remodelling at the DNA level in both human SVA and gibbon LAVA.

To assess whether slippage of the reverse transcriptase in the process of target primed reverse transcription might contribute to VNTR remodelling, we compared the VNTR sequence of LAVA_F *de novo* integrants obtained in cell-based retrotransposition assays [4] to that of their source element in the transfected vector. No differences could be observed. We consider it therefore unlikely that VNTR expansion/retraction takes place at the RNA level.

### The LAVA VNTR contains determinants of mobilization efficiency

**Figure 7:**
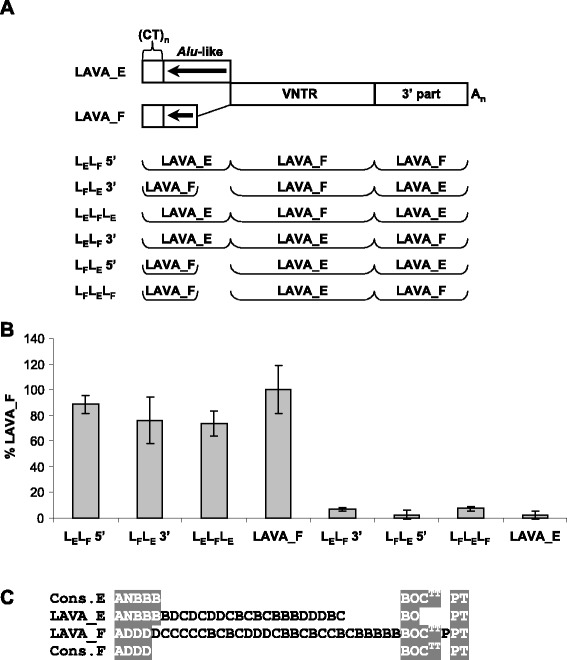
Determinants of LAVA mobilization efficiency are localized in the VNTR. **(A)** Schematic representation of the LAVA_E – LAVA_F chimeras tested. 5’ and 3’ domains of LAVA_E and LAVA_F were reciprocally exchanged at the *Alu*-like/VNTR junction and/or the VNTR/3’ part junction. Note that LAVA_F elements are characterized by a 3’ truncated *Alu*-like region. **(B)** Retrotransposition reporter assay following selection with G418. Cells were co-transfected with driver (pJM101 L1RP Δ Neo) and the respective *mneoI*-tagged chimeras or the LAVA_E/LAVA_F full-length constructs. Retrotransposition rates (+/− standard deviation, n = 3) are given relative to that of the full-length LAVA_F1 construct (100%). **(C)** Repeat unit (RU) schemata of the two LAVA elements tested. RU arrays corresponding to the respective subfamily consensus are highlighted in grey.

In a previous study we established that the structural determinants of LAVA mobilization differ from those of SVA, and that the LAVA 3’ part attenuates retrotransposition capacity [4]. In an attempt to further characterize the contribution of the different domains of LAVA elements (CT/*Alu*-like, VNTR and 3’ part) to overall mobilization efficiency, we constructed chimeras by reciprocally exchanging either the 5’ domains or the 3’ domains or both between the active LAVA_F1 and the inactive LAVA_E described previously [4]. The structure of the domain swaps is shown in Figure 7A. Quite surprisingly – against the background that major retrotransposition determinants of the other VNTR composites localize to their 5’ domains [4,8] – we found that all chimeras containing the VNTR of the active LAVA_F1 were active as well. By contrast, none of the chimeras containing the VNTR of the inactive LAVA_E was mobilized by L1 in *trans*. These results provided first evidence that determinants of efficient mobilization are localized in the VNTR domain of LAVA elements.

**Figure 8:**
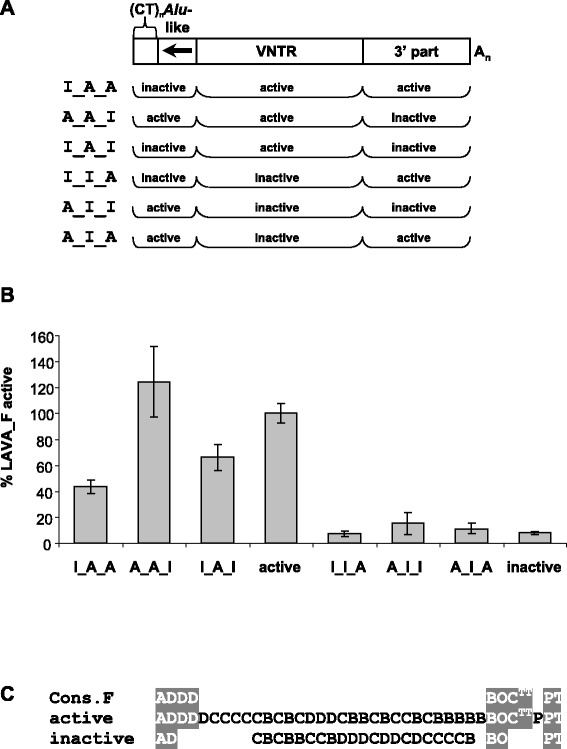
The hexameric repeat/*Alu*-like domain modulates VNTR determined LAVA mobilization capacity. **(A)** Schematic representation of the LAVA_F chimeras tested. 5’ and 3’ domains of the two LAVA_F elements were reciprocally exchanged at the *Alu*-like/VNTR junction and/or the VNTR/3’ part junction. **(B)** Retrotransposition reporter assay following selection with G418. Cells were co-transfected with driver (pJM101 L1RP Δ Neo) and the respective *mneoI*-tagged chimeras or the LAVA_F full-length constructs. Retrotransposition rates (+/− standard deviation, n = 3) are given relative to that of the full-length LAVA_F1 construct (100%). **(C)** Repeat unit (RU) schemata of the two LAVA elements tested. RU arrays corresponding to the respective subfamily consensus are highlighted in grey.

To exclude that the effect observed resulted from incompatibility between the LAVA_F1 domains (especially the truncated *Alu*-like region) and the particular 5’ structure of the LAVA_E VNTR, we initiated a second set of experiments in which domains were exchanged between an active and an inactive element of the same subfamily: LAVA_F. The structure of the chimeras and the results of the experiments are shown in Figure 8. Again, inclusion of the VNTR of the inactive element in the chimeras led to a drastic reduction in retrotransposition efficiency to the level obtained for the inactive element (Figure 8B, chimeras I_I_A, A_I_I and A_I_A). However, contrary to the results obtained for the LAVA_E/LAVA_F1 chimeras, the “LAVA_F only” chimeras containing the VNTR of the active element were mobilized at significantly different rates. Combination of the active CT/*Alu*-like/VNTR with the 3’ domain of the inactive element resulted in a retrotransposition rate comparable to that of the active element (chimera A_A_I, Figure 8). Presence of the inactive CT/*Alu*-like in chimeras I_A 5’ and I_A_I (Figure 8) resulted in a 30 to 60% reduction of the mobilization rate compared to the active element. From these findings we conclude that (i) the structure and/or length of the VNTR are crucial for efficient mobilization and (ii) the 5’ CT/*Alu*-like domain of LAVA elements modulates retrotransposition rates.

## Discussion

### SVA and LAVA VNTRs evolve at the level of structural organization

Up to date the investigation of VNTR composite evolution has been largely limited to the analysis of their 5’ CT/*Alu*-like and 3’ SINE-R (SVA)/U1-*Alu*Sz-U2-L1ME (LAVA) domains. This has been complemented by the observations that VNTR length is negatively correlated with time (i.e. younger SVA subfamilies display longer VNTRs) [5] and that in the younger subfamilies the VNTR 5’ and 3’ parts are clearly distinct with regard to their repeat unit content [10]. We now show that the VNTRs of both hominid (orangutan, chimpanzee, human) SVA and gibbon LAVA evolve along the networks established for the elements’ 3’ domains. Organization of the VNTR in terms of the arrangement of repeat units is clearly subfamily-specific and has evolved over time. Thus, sequence evolution in the elements’ 3’ parts is paralleled by structural evolution in the VNTR. VNTR composites are, to our knowledge, the only non-LTR retrotransposons harbouring complex internal tandem repeats. It is therefore difficult to draw parallels to the evolution of other class I mobile elements. Studies addressing the evolution of VNTRs across primates are scarce. Interspecies differences in length and composition have been reported e.g. for the VNTRs in the serotonin transporter gene promoter [22] and in intron 3 [23,24], in the *monoamine oxidase A* upstream regulatory region [25] and in the *dopamine receptor D4* coding region [26]. These VNTRs have been shown to be involved in transcriptional [14,15] and post-transcriptional [27] regulation. Their evolution can be assumed to be constrained by their function in regulation of gene expression. When/if SVA/LAVA VNTRs act as transcription regulatory sequences for nearby genes, the same constraints apply. An additional set of constraints on their evolution can be assumed to be imposed by their characteristics as non-autonomous retrotransposons and by the necessity to escape host surveillance in order to amplify.

### SVA and LAVA VNTRs differ in organization and complexity

LAVA elements have initially been identified based on the *Alu*-like and VNTR domains shared with SVA [2]. The analysis presented here now reveals differences between the VNTRs of LAVA and SVA. Whereas these are not evident in the respective oldest subfamilies, SVA_A and LAVA_A, from the B subfamilies onwards the two families can be clearly distinguished based on their VNTR repeat unit content and organization. In LAVA at maximum five RUs appear to be conserved between elements of the same subfamily at the 5’ end (Figure 5). In SVA, by contrast, subfamily specific 5’ arrays of between seven and fourteen RUs are discernible. In addition, SVAs of human subfamilies D to F as well as of chimpanzee SVA_PtA are characterized by internal and 3’ terminal arrays composed of one to four copies of a long RU (K or L/L’), a 41 bp G-type RU and a short C-type RU. A similar tendency of the VNTR central part made up of repeated arrays of RUs is to be observed in the younger orangutan SVA subfamilies (Figure 4A).

LAVA elements, however, lack such repeated internal arrays. It is only in the youngest LAVA subfamilies E, F0 and F, that 3’ arrays including longer (42 bp) RU types (O, P) arise. The comparison between SVA and LAVA VNTRs does, however, require qualification: the quality of the gibbon genome build from which LAVA sequences were obtained is still lower than that of the current human build. Due to their repetitive nature and the presence of repeat units identical in sequence at different positions in the VNTR, VNTRs are difficult to assemble when sequence overlap is used. A considerable number of LAVA elements still display assembly gaps. Thus, we cannot completely exclude that LAVA subfamily specific internal RU arrays exist. They are possibly “hidden” in the deficiencies of the current genome assembly.

Interestingly, in LAVA subfamilies C1, C4 and E, elements with two different RU arrays at their 5’ ends are found (Figure 5). Theoretically, the C1 and C4 elements displaying the 53 bp N-type RU could have resulted from gene conversion taking place between a LAVA_E (ANBB) and a LAVA_C1 or C4 element lacking the N-type RU. In this case the sequence of the *Alu*-like region would be expected to correspond (at least at its 3’ end) to the LAVA_E consensus. Except for a single C4 element this is not the case (data not shown). Likewise, the minority fraction of LAVA_E elements lacking the N-type RU could have been derived from another subfamily. However, both the 5’ and 3’ domains of these elements conform to the LAVA_E subfamily consensus.

### VNTR remodelling takes place at the DNA level and involves microhomologies

Our finding that remodelling of VNTRs takes place at the DNA level is in line with a recent report on the existence of allelic variants of SVA VNTRs [10,28]. Interestingly, the authors of the studies found RU copy number variation only in the 3’ part characterized by the long F and K repeat units (VNTR according to their nomenclature) and not in the 5’ part (TR according to their nomenclature) which comprises 37 to 40 bp RUs (A, B and C) only. In our test set, by contrast, RU copy number variation is found in both the 5’ and 3’ arrays (Figure 6A).

With regard to the mechanism which mediates expansion or contraction in the SVA VNTR region, Ostertag and colleagues [9] discussed “unequal homologous recombination” but cited Levinson (slipped-strand mispairing - SSM - [29]). Hancks and Kazazian [30] suggested non-allelic homologous recombination (NAHR). NAHR by unequal crossing over appears unlikely, given that the homology blocks found at the breakpoints are – with one exception – considerably shorter than the 34 bp reported as minimum length of an efficient processing segment [31]. Slipped-strand mispairing [29] or replication slippage requires that the two segments identical in sequence occur within the region expected to be single-stranded during replication. Thus, there is a limitation to the distance between the two homologous sequences – in humans of about 200 bp (the length of an Okazaki fragment). The largest indel identified in our analysis extends over 192 bp (and most likely “masks” two independent events – deletion in chimpanzee and duplication in human – element D15, Figure 6A). All other indels affect at most three RUs, in most of the cases only a single RU is lost/duplicated. Remodelling of SVA/LAVA VNTRs at the DNA level, therefore, meets the requirements for replication slippage. However, microhomologies at breakpoints have also been observed for two other mechanisms involved in the generation of copy number variation: microhomology-mediated end joining (MMEJ) and microhomology-mediated break-induced replication (MMBIR). MMEJ is frequently accompanied by the insertion of nucleotides at the breakpoint (for review on MMEJ see [32]) which we did not observe in our sample. MMBIR involves annealing of 3’ single-stranded ends to the lagging strand template of another fork (template switch). The model, however, has been developed to explain duplication/deletion events whose lengths exclude replication slippage in a single fork as the causative mechanism [33]. As all mechanisms presented have different requirements in terms of the proteins involved, it would be interesting to see what happens to retrotransposon VNTRs in test systems lacking components of the respective pathways. This, however, is beyond the scope of the current study.

### What is the function of the VNTR in retrotransposition?

In SVA complete deletion of the VNTR leads to a significant reduction in mobilization rates, whereas partial deletion results in an increase [8]. These observations permit, however, only limited conclusions on the role of the VNTR in retrotransposition. All deletion mutants tested by Hancks and colleagues [8] are characterized by a juxtaposition of sequence and structural features in a way that does not exist in nature (*Alu*-like domain/SINE-R or parts of repeat units/SINE-R). Our analysis shows that the VNTR internal structure of the younger SVA subfamilies is complex and well conserved – indicating a role for the VNTR in retrotransposition. It is tempting to speculate that the VNTR stabilizes the elements’ RNA secondary structure in a way that ensures a specific orientation of the 5’ CT/*Alu*-like domain and the 3’ SINE-R relative to each other. The internal K_1-4_GC’ arrays do not appear to affect mobilization efficiency, as all SVA elements reported to be active in the literature (H2D, H11D [8], H19_27 and H10_1 [7]) differ with respect to their presence and number (Additional file 2).

Surprisingly, we found that in LAVA the VNTR of an active element can confer retrotransposition competence when combined with the 5’ and 3’ domains of an inactive element. Thus, key determinants for efficient mobilization appear to be localized in the LAVA central domain. The data obtained for chimeras between active and inactive elements of the same subfamily (Figure 8B) point to a modulatory role of the 5’ CT/*Alu*-like region of LAVA.

Overall, these findings provide further support for the notion that the requirements for efficient mobilization differ between SVA and LAVA: in SVA key determinants reside in the 5’ hexameric repeat/*Alu*-like region [8]. LAVA retrotransposition efficiency, in contrast, seems to depend on characteristics of the VNTR. There appears to be only a modulatory function for the 5’ domain.

The comparison of the VNTRs does not provide any clues on specific structures that might characterize an active element, except for an obvious difference in length between the active LAVA_F and the inactive elements. Both LAVA_E and the active LAVA_F differ from the subfamily consensus 3’ array by one RU, whereas the inactive LAVA_F shows deviation from the subfamily consensus in both the 5’ and 3’ arrays (Figures 7C and 8C). The question of which of the internal RUs or RU arrays are critical for LAVA retrotransposition will, at least, require comparison of the VNTR across a larger number of active elements. Unfortunately, additional LAVA elements tested so far proved to be inactive as well.

### The SVA VNTR and transcriptional silencing

Recently, two independent studies [11,34] addressed the role of KRAB zinc-finger (KZNF) proteins and their cofactor TRIM28/KAP1 in transcriptional silencing of retrotransposons in human embryonic stem cells. Both found a preferential association of TRIM28/KAP1 with SVA elements. Turelli and colleagues [34] note that TRIM28/KAP1 “was significantly more associated with older family members (types A through D) than with their younger, human restricted counterparts (types E and F)”. They also state that TRIM28/KAP1-bound SVA elements contain a significantly higher number of repeat units in the VNTR. Thus, there appears to be a connection between the SVA VNTR and TRIM28/KAP1 recruitment. It will be interesting to see whether there is a correlation between different VNTR structures, as determined in our study, and TRIM28/KAP1 binding.

Jacobs and colleagues identified ZNF91 as the KZNF that recruits TRIM28/KAP1 to SVAs in the human genome [11]. Using reporter gene assays they demonstrated that the VNTR domain of a human SVA_D element is necessary and sufficient for ZNF91-mediated transcriptional repression. Interestingly, only human ZNF91 and a reconstructed hominine ZNF91 (as it probably existed in the last common ancestor of humans and gorillas) were found to efficiently repress SVA_D-driven reporter gene activity. These two ZNF91 variants differ from great ape ZNF91 (as it existed in the last common ancestor of humans and orangutans) and orangutan ZNF91, amongst others, by the presence of additional seven zinc fingers. The structural changes giving rise to hominine ZNF91 occurred 8–12 million years (Myr) ago, after the split of orangutan from the gorilla/chimpanzee/human lineage [11]. This coincides with the time of emergence of SVA subfamilies SVA_B to SVA_D (11.56, 10.88 and 9.55 Myr ago) [5]. SVA_B is the first subfamily in which longer repeat units (E, G, H, and especially I) are found in the VNTR. The repeat unit patterns established for our sample sets of SVA_B and SVA_C elements (Additional file 2) suggest that at the time of their amplification there probably existed internal amplifying RUs (or no longer recognizable arrays of RUs) comparable to those found in SVA_D to SVA_F. By contrast to the KGC’ arrays in the younger subfamilies SVA_D to SVA_F, mainly I-type RUs amplified in SVA_B and SVA_C. Testing the repressive capacity of great ape versus hominine ZNF91 with SVA_B and SVA_C elements could, possibly, establish the relative timing of structural changes in SVA VNTRs and of those in ZNF91.

Our analysis of orangutan SVAs revealed that new subfamilies emerged in this lineage. They are characterized not only by distinct sequences of their SINE-R domains, but also show subfamily-specific organization of the VNTR. Evolution of the VNTR structure in orangutan SVAs appears to be governed by the same principle as observed for hominine SVAs: organization of the central part of the VNTR in arrays of repeat units and expansion/amplification of these arrays. Once the necessary tools (orangutan pluripotent stem cells) will be available, it will be interesting to see whether and how VNTR structural evolution is linked to KZNF/KAP1-mediated control in this species.

## Conclusions

The results presented show that the central VNTR domain of LAVA and SVA evolves at the level of structural organization involving family- and subfamily-specific repeat units. Whereas in LAVA only the 5’ and 3’ ends of the domain are clearly structured, the younger SVA subfamilies are characterized by highly organized internal amplifying RU arrays. The composition of these arrays differs between the orangutan and chimpanzee/human lineages. The lineage-specific differences observed in the central domain are likely to influence the interaction of LAVA and SVA with host factors regulating their expression and mobilization. Our observations therefore provide a starting point for further investigations aiming to explain and understand the differences in amplification dynamics of VNTR composite retrotransposons across hominoids.

## Methods

### Sequence analysis – datasets

The datasets used in the analysis are summarized in Table 1.

#### SVA2_MMU_ and SVA2_NLE_

Thirty elements each were retrieved from the *Macaca mulatta* (MMU) and *Nomascus leucogenys* (NLE) genomes by BLAT [35,36] using the SVA2 3’ unique sequence as query.

#### SVA_NLE_, PVA, FVA

The datasets are those described previously [4]. In case of PVA only elements displaying a 5’ complete VNTR (i.e. containing at least the 3’ part of the *Alu*-like region) were analyzed.

#### LAVA

Depending on the size of the subfamily 5, 10 or 20 elements (corresponding to 5-10% of the number of subfamily members) were randomly selected from the set of LAVA elements described in Carbone et al. [37]. Only elements displaying a 5’ complete VNTR (i.e. containing at least the 3’ part of the *Alu*-like region) were considered. Where possible, elements containing assembly gaps in the VNTR region were excluded. In some subfamilies, however, exclusion of such elements would have led to a number too small to be analyzed. In these cases (e.g. LAVA_C1) also elements containing assembly gaps were taken into account. To establish the distribution of the N-type 53 bp repeat unit (RU) among the elements of the younger subfamilies, the first three 5’ RUs of all elements from LAVA_C to LAVA_E were analyzed.

#### SVA_HSA_ and SVA_D shared between humans and chimpanzee

Human SVA elements (SVA_HSA_) were retrieved using the repeatmasker pre-masked genome section [38] and the UCSC genome browser [36]. Ten full-length elements were analyzed per subfamily. In case of SVA_D ten additional elements shared between humans and chimpanzee were examined. Chimpanzee orthologs were obtained using the UCSC genome browser.

#### Chimpanzee specific SVA elements

VNTR full-length chimpanzee specific SVA elements were retrieved using the repeatmasker pre-masked genome section [38] and the UCSC genome browser [36]. Alignment of the SINE-R of a total of 294 elements identified a subfamily of 83 SVAs clearly different from SVA_D. We assume that these elements are representatives of the SVA_PtA subfamily reported by Wang and colleagues [5].

#### Orangutan SVAs

SVA elements in the *Pongo abelii* (PA) genome (GenBank Assembly ID: GCA_000001545.3) were identified using a locally implemented version of RepeatMasker [38] and the RepBase [18,19] *Homo sapiens* subfamily consensus library. Results contained in the out files corresponding to each chromosome were filtered using “in-house” R scripts in order to keep only sequences having a total length of at least 300 bp and being truncated by no more than 50 bp at their 3’ ends, relative to the consensus. Based on the filtered results, 1365 SVA sequences were retrieved by either trimming or extending the 5’ ends of each hit so that all retrieved sequences would have a total length of 600 bp.

MAFFT [39] was used to align the extracted hits with the consensus sequences of the human SVA subfamilies. The alignment was further manually curated and all sequences containing assembly gaps or major truncations were removed. A total of 119 hits corresponding to SVAs lacking the 5’ part of the SINE-R were easily identifiable and analyzed separately. The remaining 1009 sequences were subjected to subfamily analysis using COSEG [40] with default settings.

VNTR analysis was performed for five to twenty elements per subfamily, depending on subfamily size. In case of the younger subfamilies SVA_PA__7 to SVA_PA__11 5’ truncated elements and elements with assembly gaps had to be taken into account to obtain consensus VNTR schemes with sufficient support.

#### Sequence analysis, derivation of repeat unit consensus sequences and annotation

The sequences of the VNTR regions were manually split into repeat units, using the annotation provided by Ostertag et al. [9] as guideline. The entire complement of RUs obtained for a family/subfamily was then sorted first by length and further on, if applicable, by sequence – yielding a set of 19 RU types and a number of sequence variants. Consensus sequences were generated for each of the RU types/sequence variants using a majority rule approach. Sorting and consensus generation were carried out using BioEdit. Subsequently, a “repeat scheme” was established for each of the elements analyzed. A consensus for conserved arrays of RUs was then obtained by comparison of these schemata across elements within a family/subfamily. The repeat schemata for all elements analyzed (except SVA2_MMU_ and SVA2_NLE_ which were used for identification of ancestral RUs only) are provided in Additional file 2, Additional file 3, Additional file 4. In case of orangutan only the four youngest subfamilies are shown.

#### Network construction

For human SVA subfamilies A to F the network published by Wang and colleagues [5] was used. For generation of the network of orangutan SVA subfamilies the consensus sequences resulting from the COSEG analysis were combined with the consensus sequence obtained from separate analysis of the elements carrying a 5’ truncated SINE-R. The network used for LAVA is based on the subfamilies obtained from manual sorting of the LAVA set described in Carbone et al. [37]. Consensus sequences of the LAVA 3’ part (U1-*Alu*Sz-U2-L1ME) used for network construction are provided in Additional file 1: Figure S4. Median joining network analysis was performed using Network 4.6.1.2. [41] with default settings.

#### Plasmid constructs

All test vectors are based on pCEPNeo [7]. The LAVA_E and LAVA_F1 elements combined in the first set of chimeras are those described previously [4]. The inactive LAVA_F element used in the second set of chimeras was amplified from *Nomascus leucogenys* genomic DNA (kindly provided by Christian Roos, Gene Bank of Primates at the German Primate Centre, Göttingen) using primers L992_FW 5’-TTCCTCCTTTACCTCTTTTCACC-3’ and L992_REV 5’-GCTCTGTAGTGCTTACTGCCATC-3’ and Phusion Hot Start II (Thermo Scientific) according to the manufacturer’s instructions. DMSO was added to the reaction to a final concentration of 3% and denaturation time was extended to 30 seconds. The amplified element was subcloned into pJET 1.2 (Thermo Scientific). Re-amplification was carried out using primers L992_Kpn 5’-AC*GGTACC*AGCTGTGCTCACTGTTTTGC-3’ and L286_Nhe 5’-AG*GCTAGC*GCACACAAAAACAATAAACATTTTCTAA-3’. The reamplification product was subcloned again into pJET 1.2 for sequencing and further cloning. Finally, the element was transferred into pCEPNeo via KpnI/NheI. An alignment of the amplified sequence to the reference genome sequence is provided in Additional file 1: Figure S7 A.

Chimeric elements were inserted into pCEPNeo via KpnI/NheI. All amplification and cloning steps were verified using Sanger sequencing. The fine structure of the junctions between the domains is shown in Additional file 1: Figure S7 B.

#### L_E_L_F_ 5’ and L_F_L_E_ 5’

For generation of the L_E_L_F_ 5’ chimera the LAVA_E CT-*Alu*-like region and the LAVA_F1 VNTR/3’ end were combined in pCEPNeo using KpnI/BstAPI(blunt)/AvaI(blunt)/NheI. The L_F_L_E_ 5’ domain swap was generated by amplification of the LAVA_F1 5’ end using a downstream primer with a SmaI recognition site (L284_Kpn 5’-AC*GGTACC*TAGAAGTAGAAAACACCGAC-3’; L284_Sma 5’-AT*CCCGGG*CTCGGGAGGCTGAG-3’). The amplification product was then combined with the LAVA_E VNTR/3’ end and cloned into pCEPNeo using KpnI/SmaI/BstAPI(blunt)/NheI.

#### L_E_L_F_ 3’ and L_F_L_E_ 3’

For generation of the L_E_L_F_ 3’ and L_F_L_E_ 3’ chimeras the 3’ ends of the LAVA_E and LAVA_F1 elements were amplified using an upstream primer providing an RsaI recognition site (LA_E_Rsa 5’-GT*GTAC*CACCGAGGCCAGAAGCAATG-3’; LA_F_Rsa 5’- GT*GTAC*CATGGAGGCCA GAAGCAATG-3’) and an NheI recognition site containing downstream primer (L876_Nhe5’-AG*GCTAGC*GCACACAAAAACAATAAACATTTTCTAA-3’). The elements’ CT-*Alu*-like-VNTR domains were then reciprocally combined with the 3’ ends in pCEPNeo using KpnI/AccI(blunt)/RsaI/NheI.

#### L_E_L_F_L_E_ and L_F_L_E_L_F_

Chimeras L_E_L_F_L_E_ and L_F_L_E_L_F_ were obtained by reciprocally combining the CT-*Alu*-like-VNTR domains of L_E_L_F_ 5’ and L_F_L_E_ 5’ with the RsaI amplified 3’ ends in pCEPNeo using KpnI/AccI(blunt)/Rsa/NheI.

#### A_I_I and I_A_A

For generation of the two 5’ chimeras A_I_I and I_A_A the 5’ CT-*Alu*-like regions of the elements were combined in pCEPNeo with the VNTR/3’ ends of the respective other element using KpnI/AvaI/NheI.

#### A_A_I and I_I_A

In case of the A_A_I chimera the 3’ reciprocal exchange was achieved by combining the CT-*Alu*-like/VNTR region of the active element LAVA_F1 element [4] with the 3’ end of the inactive element (see above) in pCEPNeo using KpnI/MbiI/NheI. To obtain the I_I_A 3’ chimera, the CT-*Alu*-like/VNTR region of the inactive element was combined in pCEPNeo with the RsaI amplified 3’ end (see L_E_L_F_ 3’) of the active element using KpnI/AccI(blunt)/Rsa/NheI.

#### A_I_A and I_A_I

Chimera A_I_A was obtained by combining the CT-*Alu*-like-VNTR domain of chimera A_I_I with the RsaI amplified 3’ end (see L_E_L_F_ 3’) of the active element using KpnI/AccI(blunt)/Rsa/NheI. Chimera I_A_I was generated by combining the CT-*Alu*-like-VNTR domain of chimera I_A_A with the 3’ end of the inactive element in pCEPNeo using KpnI/MbiI/NheI.

#### Tissue culture and retrotransposition assays

HeLa HA cells (kindly provided by J. Moran and previously shown to support detectable levels of SVA retrotransposition) [7] were cultured in DMEM (Lonza) 4.5 g/l Glucose, 10% FCS. Cell-based assays to assess retrotransposition in *trans* were carried out as described previously [7,42] with minor modifications. Briefly, 4x10^5^ cells were seeded on T25 flasks 24 hours before transfection. They were then co-transfected with 2 μg test plasmid and 2 μg L1 expression vector (pJM101 L1RPΔNeo) or pCEP4 (Invitrogen), respectively, using X-tremeGENE 9 (Roche) according to the manufacturer’s instructions. The medium was changed 24 h post transfection and cells were re-seeded 48 h post transfection. G418 selection was initiated 72 h post transfection and continued for 12 days. Subsequently, cells were stained with Giemsa (Merck) and colonies were counted.

### Availability of supporting data

The data sets supporting the results of this article are included within the article and its additional files.

## Additional files

Additional file 1: Figure S1. Sequence evolution within repeat unit (RU) types across SVA2/SVA_NLE/HSA_/PVA/FVA (A) and LAVA (B) families/subfamilies. Alignments of consensus sequences generated for the respective RU type in the families/subfamilies are shown. SVA_A to SVA_F denote human SVA subfamilies. **Figure S2.** Position specific sequences of RUs in the younger human and chimpanzee (A) and orangutan (B) SVA subfamilies. Alignments of consensus sequences generated for the RUs at specific positions are shown. Chimpanzee RUs are denoted with Pt. MMU – *Macaca mulatta*, NLE – *Nomascus leucogenys*, HSA – *Homo sapiens.* **Figure S3.** Orangutan SVA subfamilies and their phylogenetic relationships. (A) Multiple alignment of the consensus sequences obtained for the SINE-R part of orangutan SVA subfamilies. Note that the consensus of SVA_PA_1 is identical to that of human SVA_A. (B) Median joining network constructed based on the consensus sequences shown in (A). Numbers indicate the number of substitutions separating the subfamilies. Short junctions not annotated represent one substitution. SVA_PA_12 is characterized by deletion of the 5’ part of the SINE-R. **Figure S4.** Multiple alignment of the consensus sequences of the LAVA subfamilies’ 3’ parts (U1-*Alu*Sz-U2-L1ME). Subfamilies were obtained by manual sorting. The alignment shown was used for generation of the network presented in figure 5. *M. mulatta* and *N. leucogenys* represent the source sequence of the LAVA 3’ part in *Hydroxysteroid (17-beta) dehydrogenase 3* (*HSD17B3*) intron 2. **Figure S5.** Alignments of the VNTR regions of orthologous SVA_D elements in human (HSA) and chimpanzee (PTR). Microhomologies flanking breakpoints are highlighted in yellow. Repeat unit (RU) types are annotated on top of the sequences. Variant RUs in chimpanzee are indicated in red below the respective RU. Changes in the repeat unit patterns are given below the respective alignment. **Figure S6.** Alignments of the VNTR regions of *Nomascus leucogenys* LAVA elements that have amplified as parts of segmental duplications. Microhomologies belonging to the same breakpoints are highlighted in identical color. Repeat unit (RU) types are annotated on top of the sequences. Position information for the elements is provided in Additional file 3. **Figure S7.** A Alignment of the “inactive” LAVA_F element amplified from *Nomascus leucogenys* genomic DNA to the reference sequence (GGSC Nleu3.0/nomLeu3; chr9:35,010,156-35,012,794). Target Site Duplications are highlighted in red. B Fine structure of the domain junctions in LAVA chimeras. Additional file 2:
**VNTR repeat unit (RU) schemata of human, chimpanzee and orangutan SVA elements.** Conserved sequences of repeat units at the 5’ and 3’ ends of the VNTR are highlighted in yellow for SVA_A to SVA_C; internal conserved RU arrays in SVA_D to SVA_F are boxed. RUs with position specific sequences are coloured in SVA_D to SVA_F, SVA_PtA and the orangutan SVAs. RUs that could not be assigned to one of the RU types are denoted with a question mark. Additional file 3:
**VNTR repeat unit (RU) schemata of** ***Nomascus leucogenys*** **LAVA elements.** Conserved sequences of repeat units at the 5’ and 3’ ends of the VNTR are highlighted in yellow. RUs that could not be assigned to one of the RU types are denoted with a question mark.SegDup: LAVA elements amplified as part of segmental duplications. Additional file 4:
**VNTR repeat unit (RU) schemata of** ***Nomascus leucogenys*** **PVA, SVA and FVA elements.**

## Abbreviations

VNTR: Variable Number of Tandem Repeats
SINE-R: SINE of retroviral origin
PTGR: Prostaglandin reductase
FRAM: Free Right *Alu* Monomer
RU: Repeat unit
TR: Tandem Repeat
MMU: *Macaca mulatta*
NLE: *Nomascus leucogenys*
HSA: *Homo sapiens*
PA: *Pongo abelii*
Pt: *Pan troglodytes*
SSM: Slipped-strand mispairing
NAHR: Non-allelic homologous recombination
MMEJ: Microhomology-mediated end joining
MMBIR: Microhomology-mediated break-induced replication
Myr: Million years
