# Supplementary material for: Lineage specific evolution of the VNTR composite retrotransposon central domain and its role in retrotransposition of gibbon LAVA elements
Source: BMC Genomics. 2015 May 16;16(1):389. doi: 10.1186/s12864-015-1543-z (PMC4432496; doi:10.1186/s12864-015-1543-z)
Supplement: Additional file 1: Figure S1. — Sequence evolution within repeat unit (RU) types across SVA2/SVANLE/HSA/PVA/FVA (A) and LAVA (B) families/subfamilies. Alignments of consensus sequences generated for the respective RU type in the families/subfamilies are shown. SVA_A to SVA_F denote human SVA subfamilies. Figure S2. Position specific sequences of RUs in the younger human and chimpanzee (A) and orangutan (B) SVA subfamilies. Alignments of consensus sequences generated for the RUs at specific positions are shown. Chimpanzee RUs are denoted with Pt. MMU – Macaca mulatta, NLE – Nomascus leucogenys, HSA – Homo sapiens. Figure S3. Orangutan SVA subfamilies and their phylogenetic relationships. (A) Multiple alignment of the consensus sequences obtained for the SINE-R part of orangutan SVA subfamilies. Note that the consensus of SVA_PA_1 is identical to that of human SVA_A. (B) Median joining network constructed based on the consensus sequences shown in (A). Numbers indicate the number of substitutions separating the subfamilies. Short junctions not annotated represent one substitution. SVA_PA_12 is characterized by deletion of the 5’ part of the SINE-R. Figure S4. Multiple alignment of the consensus sequences of the LAVA subfamilies’ 3’ parts (U1-AluSz-U2-L1ME). Subfamilies were obtained by manual sorting. The alignment shown was used for generation of the network presented in figure 5. M. mulatta and N. leucogenys represent the source sequence of the LAVA 3’ part in Hydroxysteroid (17-beta) dehydrogenase 3 (HSD17B3) intron 2. Figure S5. Alignments of the VNTR regions of orthologous SVA_D elements in human (HSA) and chimpanzee (PTR). Microhomologies flanking breakpoints are highlighted in yellow. Repeat unit (RU) types are annotated on top of the sequences. Variant RUs in chimpanzee are indicated in red below the respective RU. Changes in the repeat unit patterns are given below the respective alignment. Figure S6. Alignments of the VNTR regions of Nomascus leucogenys LAVA elements that have amplified as [file 12864_2015_1543_MOESM1_ESM.pdf]

Figure S1

Sequence evolution within repeat unit (RU) types across SVA2/SVA$_{NLE/HSA}$/PVA/FVA (A) and LAVA (B) families/ subfamilies. Alignments of consensus sequences generated for the respective RU type in the families/ subfamilies are shown. SVA_A to SVA_F denote human SVA subfamilies.
MMU – *Macaca mulatta*, NLE – *Nomascus leucogenys*, HSA – *Homo sapiens*

A

A-type RUs

```
SVA2_MMU_A   CCTCTGCCCGGCCGCCGCACCGTCTGGGAAGTGAGGAGCG
SVA2_NLE_A   ........................................
SVA_NLE_A    ................A.C......A..............
PVA_A        ................A.C......A..............
FVA_A        ..................C..........G...G......
SVA_A_A_5'   ................A.C......A..............
SVA_B_A_5'   ................A.C.....................
SVA_C_A_5'   ................A.C.....................
SVA_D_A_5'   ................A.C.....................
SVA_E_A_5'   ................A.C.....................
SVA_F_A_5'   ................A.C...................Y.
SVA_A_A      ................A.C.....................
SVA_B_A      ................A.C.....................
SVA_C_A      ............M...A.C.....................
SVA_D_A      ............M...A.C..A..................
SVA_E_A      ................A.C.....................
SVA_F_A      ....Y.......A...A.C....................V
```

C-type RUs

```
SVA_NLE_C    CCTCTGCCCGGCCGCCCCGTCTGGGAAGTGAGGAGCG
PVA_C        .....................................
FVA_C        ..........................T..........
SVA_A_C      .....................................
SVA_B_C      ....................................C
SVA_C_C      .....................................
SVA_D_C      .....................................
SVA_E_C      .................H...................
SVA_F_C      .....................................
SVA_D_C'     ..................TA................C
SVA_E_C'     ..................TA................C
SVA_E_C''    ..................TA..............-.C
SVA_F_C'     ..................TA................C
```

E- and G-type RUs

```
SVA_B_E      TCTCCGCCCGG-CAGCCGCCCCGTCCGGGAGGTGGGGGGCAG
SVA_C_E      ...........-..............................
SVA_B_G      C.C.Y......C............................-.
SVA_C_G      C.C........C.........Y..................-.
SVA_D_G      C.C........C......................A.....-.
SVA_E_G      C.C........C......................A.....-.
SVA_F_G      C.C........C......................A.....-.
```

H- and I-type RUs

```
SVA_B_H      CCCCCGCCCGGCCAGCCRCCCCGTCCGGGAGG----TGGGGGGCAG
SVA_C_H      ..T.....T.......................----.......GC.
SVA_B_I      ................................GAGG..........
SVA_C_I      ................................GAGG..........
```

F-, K- and L-type RUs

```
SVA_D_F      TCTCCGCCCGGCA-GCCACCCCGTCCGGGAGGGAGGTGGGGGGGTCAGC
SVA_E_F      .............-....................................
SVA_F_F      .............-....................................
SVA_D_K      C.C.........CA...G..........................-.....
SVA_E_K      C...T.......CA...G..........................-.....
SVA_F_K      C.C.........CA...G..........................-.....
SVA_E_L'     --------....CA...G..........................-.....
SVA_E_L      C...T.......CA...G..................C........GGGT
```

B

A-type RUs

```
LAVA_A2_A5'     CCTCTGCCCGGCCGCCGCCCCGTCTGGGAGGTGGGGAGCG
LAVA_A1_A5'     .......YS............R..................
LAVA_B1A_A5'    ........................................
LAVA_B1D_A5'    ..................T.....................
LAVA_B1R_A5'    ........................................
LAVA_B1B_A5'    ........................A...............
LAVA_B1M_A5'    ........................................
LAVA_B2R2_A5'   ........................................
LAVA_B2E_A5'    ........................................
LAVA_B2R1_A5'   ........................................
LAVA_B2A_A5'    ........................................
LAVA_B2C_A5'    ........................................
LAVA_B1G_A5'    ........................................
LAVA_B1L_A5'    ..C.....................................
LAVA_B1F_A5'    ..C.....................................
LAVA_C1_A5'     ..C.....................................
LAVA_C2_A5'     ..C........T.....................A......
LAVA_C4_A5'     ..C.....................................
LAVA_D_A5'      ..C..............................A......
LAVA_E_A5'      ..C.....................................
LAVA_F0_A5'     ..C.....................................
LAVA_F_A5'      ..C..........A..........................
LAVA_A2_A       .........................A...A...A......
LAVA_A1_A       ...S.....R...R..A........A...A...A......
LAVA_B1A_A      ................A........A...A...A......
LAVA_B1D_A      .........A......A........A...A...A......
LAVA_B1R_A      ................A........A...A...A......
LAVA_B1B_A      ................R................A......
LAVA_B1M_A      ................A........A...A...A......
LAVA_B2R2_A     ................A........A...A...A......
LAVA_B2E_A      ................A........A...A...A......
LAVA_B2R1_A     ................A........A...A...A......
LAVA_B2A_A      ................A........A...A...A......
LAVA_B2C_A      ................A........A...A...A......
LAVA_B1G_A      ................A........A...A...A......
LAVA_B1L_A      .........R......A........A...A...A......
```

C-type RUs

```
LAVA_A2_C       CCTCTGCCCGGCCGCCCCGTCTGGGAAGTGAGGAGCG
LAVA_A1_C       .....................................
LAVA_B1A_C      .....................................
LAVA_B1D_C      .....................................
LAVA_B1R_C      .....................................
LAVA_B1B_C      .....................................
LAVA_B1M_C      .....................................
LAVA_B2R2_C     .....................................
LAVA_B2R1_C     .....................................
LAVA_B2E_C      .....................................
LAVA_B2A_C      .....................................
LAVA_B2B_C      .....................................
LAVA_B2C_C      .....................................
LAVA_B1G_C      .....................................
LAVA_B1L_C      .....................................
LAVA_B1F_C      .....................................
LAVA_C1_C       .....................................
LAVA_C2_C       .....................................
LAVA_C4_C       .....................................
LAVA_D_C        .....................................
LAVA_E_C        .....................................
LAVA_F0_C       .....................................
LAVA_F_C        .....................................
LAVA_B2B_C_5'   .............A......................A
LAVA_C2_C_C2    .G...A.....T.....T...................
LAVA_B1A_C_T    .............R............T...G......
LAVA_B1R_C_T    ..........................T...G.....R
LAVA_B1M_C_T    ..........................T...G......
LAVA_B1G_C_T    ..................R.......T...G......
LAVA_B1L_C_T    ........H.................T...G......
LAVA_B1F_C_T    ............T.............T...G......
LAVA_B2R2_C_T   ...Y....Y.................T...G....Y.
LAVA_B2E_C_T    ........T.........A.......T...G......
LAVA_B2R1_C_T   ........T.........A.......T...G......
LAVA_B2A_C_T    ........T.................T...G......
LAVA_B2C_C_T    ........T.................T...G......
LAVA_C1_C_T     ..........................T...G......
LAVA_C2_C_T     ..........................T...G......
LAVA_C4_C_T     ..........................T...G......
LAVA_D_C_T      .............R............T...G......
LAVA_C2_C_TT    T.......T............................
LAVA_C4_C_TT    T.......T............................
LAVA_D_C_TT     T.......T........G...................
LAVA_E_C_TT     T.......T............................
LAVA_F0_C_TT    T.......T............................
LAVA_F_C_TT     T.......T............................
```

B - continued

D-type RUs

```
LAVA_B1A_D     CCTCTGCCCGGCCGCCCCGTCTGGGAAGAAGTGAGGAGCG
LAVA_B1D_D     ........................................
LAVA_B1R_D     ........................................
LAVA_B1B_D     ........................................
LAVA_B1M_D     ........................................
LAVA_B2R2_D    ........................................
LAVA_B2E_D     ........................................
LAVA_B2R1_D    ........................................
LAVA_B2A_D     T......................................R
LAVA_B2B_D     ........................................
LAVA_B1G_D     ........................................
LAVA_B1L_D     ........................................
LAVA_B1F_D     ........................................
LAVA_C1_D      ........................................
LAVA_C2_D      ........................................
LAVA_C2_D_C2   T.............................A....C.A..
LAVA_C4_D      ........................................
LAVA_D_D       .....................C..................
LAVA_E_D       .....................C..................
LAVA_F0_D      .....................C..................
LAVA_F_D       .....................C..................
```

N-type RUs

```
LAVA_C1_N   TCTCTGCTCGGCCGCCCCTCTGCCCGGCCGCCCCGTCTGGGAAGTGAGGAGCG
LAVA_C4_N   .............................T....A..................
LAVA_E_N    .......C.....................T....A..................
```

O-type RUs

```
LAVA_E_O    CCTCTGCCCGGCCACCTATCGTCTGGGAAGAAGTGAGGAGCG
LAVA_F0_O   ................C.........................
LAVA_F_O    ..........................................
```

P-type RUs

```
LAVA_E_P    CCTCTGCCCGGCCGCCCCGTGTCTGGGAAGAAGTGAGGAGCG
LAVA_F0_P   ..........................................
LAVA_F_P    ..........................................
```

Figure S2 **Position specific sequences of RUs in the younger human and chimpanzee (A) and orangutan (B) SVA subfamilies.** Alignments of consensus sequences generated for the RUs at specific positions are shown. Chimpanzee RUs are denoted with Pt.

A

A-type RUs

```
SVA_D_A1    CCTCTGCCCGGCCGCCACCCCGTCTGGGAAGTGAGGAGCG
SVA_E_A1    ........................................
SVA_F_A1    ......................................Y.
SVA_Pt_A1   ........T...A...........................
SVA_D_A2    T................T...A...A..............
SVA_E_A2    T................T...A...A.....C........
SVA_F_A2    T...C............T...A...A..............
SVA_Pt_A2   T................T...A...A..............
SVA_D_A3    .....T...........T...A...A..............
SVA_E_A3    .....T...C.......T...A...A..............
SVA_F_A3    .....T...R.......T.A.A...A..............
SVA_Pt_A3   .....T...........T.A.A..................
SVA_D_A4    ...............G.............G..........
SVA_E_A4    .......TG......A....T........G..........
SVA_F_A4    ..............AG.............G..........
SVA_D_A5    ........T...AA..G...Y.....A............C
SVA_E_A5    ........T...AA..G.........A............C
SVA_F_A5    ........Y...AA............A............C
SVA_Pt_A4   ........T...AA..G.........A............C
SVA_D_A6    ....C.......A...G.........A............C
SVA_E_A6    ....C.T.....A...........................
SVA_F_A6    ....C.......A..TG.........A............C
SVA_Pt_A5   ....C.......A.............A.............
SVA_D_A7    ....C.......A...........................
SVA_F_A7    T...C.......A........A..................
```

B-type RUs

```
SVA_D_B_1   TCTCTGCCTGGCCGCCCATCGTCTGGGATGTGAGGAGCC
SVA_E_B_1   .............................A.........
SVA_F_B_1   .......................................
SVA_Pt_B_1  ....C.....A............................
SVA_D_B_2   ........C................A......G.....G
SVA_E_B_2   ........C................A......G.....A
SVA_F_B_2   ........C................A......G.....G
SVA_Pt_B_2  ........C................A......G.....A
```

C-type RUs

```
SVA_D_C1    CCTCTGCCTGGCTGCCCAGTCTGGAAAGTGAGGAGCG
SVA_E_C1    .....................................
SVA_F_C1    .....................................
SVA_Pt_C1   .....................................
SVA_D_C2    ........CC..C....C......G.T..........
SVA_E_C2    ........CC..C....T......G.T..........
SVA_F_C2    ........CC..C....CA.....G.T..........
SVA_Pt_C2   ........CT..C....C...C..G.T..........
SVA_D_C3    T.......C...C....C.....AG..........AC
SVA_E_C3    T.......C...C....C.....AG.........AAC
SVA_F_C3    T.......C...C....C.....AG..........AC
SVA_Pt_C3   T.......C...C....T.....AG..........AC
SVA_D_C'    ........C...C....CTA....G...........C
SVA_E_C'    ........C...C....CTA....G...........C
SVA_F_C'    ........C...C....CTA....G...........C
SVA_Pt_C'   ........C...C....CTA....G...........C
SVA_E_C_T   .T......C...C....CTA....G...........C
```

B

A-type RUs

```
SVA_Pa_7_A1     CCTCTGCCCCACCGCCACCCCGTCTAGGAAGTGAGGAGCG
SVA_Pa_8_A1     ..........G............................A
SVA_Pa_9_A1     ..........G............................A
SVA_Pa_10_A1    ..........G................A...........A
SVA_Pa_11_A1    ..........G.........Y..................A
SVA_Pa_7_A2     ........TGG..............G..............
SVA_Pa_8_A2     ........TGG.....G........G...G..........
SVA_Pa_9_A2     ........TGG..............G..............
SVA_Pa_10_A2    ........TGG.........T....G.............R
SVA_Pa_11_A2    ........TGG..............GA.............
SVA_Pa_7_A3     .........GG.T........A.A.G..............
SVA_Pa_8_A3     .........GG.T........A.A.G..............
SVA_Pa_9_A3     .........GG.T........A.A.G..............
SVA_Pa_10_A3    .........GG.T........A.A.G..............
SVA_Pa_11_A3    .........GG.T........A.A.G..............
SVA_Pa_7_A_3'   .........AG.......A......G..............
SVA_Pa_8_A_3'   .........AG.......A......G..............
SVA_Pa_9_A_3'   .........AG.......A......G..............
SVA_Pa_11_A_3'  .........AG.......A......G..............
```

Q-type RUs

```
SVA_Pa_7_Q      CCTCTGCCTGGCCGCCACCCCGTCTGGGAGGAAGTGAGGAGCG
SVA_Pa_8_Q      ........C..................................
SVA_Pa_9_Q      ........C..................................
SVA_Pa_10_Q     ........C..................................
SVA_Pa_11_Q     ...........................................
SVA_Pa_7_Q_3'   ........C...T.......G............C.........
SVA_Pa_8_Q_3'   ........C...T.......G..A...................
SVA_Pa_9_Q_3'   ........C...T.......G......................
SVA_Pa_10_Q_3'  ........C...T.......G......................
SVA_Pa_11_Q_3'  ........C...T.......G......................
```

B-type RUs

```
SVA_Pa_7_B1     TCTCTGCCTGGCCGCCCATCGTCTGGGATGTGAGGAGCC
SVA_Pa_8_B1     .......................................
SVA_Pa_9_B1     ....................................R..
SVA_Pa_10_B1    ....................................A..
SVA_Pa_11_B1    .......................................
SVA_Pa_7_B2     C.......C...........A.................G
SVA_Pa_8_B2     C.......C.............................G
SVA_Pa_9_B2     C.....................................G
SVA_Pa_10_B2    C.......C.............................G
SVA_Pa_11_B2    C.......C...........A.................G
```

C-type RUs

```
SVA_Pa_7_C1     CCTCTGCCCGGCCGCCCTATCTGGGAAGTGAGGAGCG
SVA_Pa_8_C1     .....................................
SVA_Pa_9_C1     .....................................
SVA_Pa_10_C1    .....................................
SVA_Pa_11_C1    .....................................
SVA_Pa_7_C      .................CG.......G..........
SVA_Pa_8_C      .................CG.......G..........
SVA_Pa_9_C      .................CG.......G..........
SVA_Pa_10_C     .................CG..................
SVA_Pa_11_C     .................CG.......V..........
```

R-type RUs

```
SVA_Pa_7_R        CCTCTGCCCGGGCGGCCCCGTCTGGGAAGCGAGGAGCG
SVA_Pa_8_R1       ........T....................T........
SVA_Pa_8_R2       ......................................
SVA_Pa_9_R        ......................................
SVA_Pa_10_R       ......................................
SVA_Pa_11_R       ......................................
SVA_Pa_11_R_3'    ................T.....G......T........
```

Figure S3 Orangutan SVA subfamilies and their phylogenetic relationships. (A) Multiple alignment of the consensus sequences obtained forthe SINE-R part of orangutan SVA subfamilies. Note that the consensus of SVA_PA_1 is identical to that of human SVA_A. (B) Median joining network constructed based on the consensus sequences shown in (A). Numbers indicate the number of substitutions separating the subfamilies. Short junctions not annotated represent one substitution. SVA_PA_12 is characterized by deletion of the 5' part of the SINE-R.

A

```
SVA_PA_1  CAGCTCCGAAGAGACAGCGACCATCGAGAACGGGCCATGATGACGATGGCGGTTTTGTCGAAAAGAAAAGGGGGAAATGTGGGGAAAAGAAAGAGAGATCAGATTGTTAC
SVA_PA_2  ..........................................................T...................................................
SVA_PA_3  ..........................................................T...................................................
SVA_PA_4  ..........................................................T...................................................
SVA_PA_5  ..........................G..G..........G.................T........GG.......G..........G...G................G.
SVA_PA_6  ..........................G..GT.........G.................T........GG.......G......A...GA..G................G.
SVA_PA_7  ..........................G..G..........G.................T...G....GG..A....G..........G...G................G.
SVA_PA_8  ..........................G..G..........G.................T...G....GG.......G..........G...G................G.
SVA_PA_9  ..........................G..G..........G.................T....G...GG.......G..........G...G................G.
SVA_PA_10 ..........................G..G.C........G.................T....G...GG.......G..........G...G................G.
SVA_PA_11 ..........................G..G..........G..T..............T...G....GG..A....G..........G...G................G.
SVA_PA_12 --------------------------------------------------------------------------------------------------------------

SVA_PA_1  TGTGTCTGTGTAGAAAGAAGTAGACATAGGAGACTCCATTTTGTTCTGTACTAAGAAAAATTCTTCTGCCTTGGGATGCTGTTAATCTATAACCTTACCCCCAACCCCGT
SVA_PA_2  .......................G.............................G..G..........................G......GG....G......G......
SVA_PA_3  .......................G.............................G..G..........................G......GG....G......G......
SVA_PA_4  .......................G.............................G..G..........................G......GG....G......G......
SVA_PA_5  ..................G..G.G........................-....G..G..........................G......GG....T......G......
SVA_PA_6  ..................G..G.G........................-....G..G..........................G......GG....T......G......
SVA_PA_7  .................GG..G.G........................-....G..G..........................G......GG....T......G....C.
SVA_PA_8  ..................G..G.G........................-....G..G..........................G......GG....T......G....A.
SVA_PA_9  .................GG..G.G........................-....G..G..........................G......GG....T......G....A.
SVA_PA_10 .................GG..G.G.........T..............-....G..G..........................G......GG....T......G....A.
SVA_PA_11 .................GG..G.G........................-....G..G..G.......................G....CCGG....T......G......
SVA_PA_12 ----------------------------------------------------.G..G..........................G......GG....G......G......

SVA_PA_1  GCTCTCTGAAACATGTGCTGTGTCAACTCAGGGTTAAATGGATTAAGGGCGGTGCAAGATGTGCTTTGTTAAACAGATGCTTGAAGGCAGCATGCTCGTTAAGAGTCATC
SVA_PA_2  ..............................................................................................................
SVA_PA_3  .................................................................................................T............
SVA_PA_4  .................................................................................................C............
SVA_PA_5  .................................................................................................T............
SVA_PA_6  .................................................................................................T............
SVA_PA_7  .................................................T...............................................T............
SVA_PA_8  ..............A..................................................................................T............
SVA_PA_9  ..............A..................................................................................T............
SVA_PA_10 ..............A...............................................A..................................T............
SVA_PA_11 ............................................................................G....................T............
SVA_PA_12 .................................................................................................T............

SVA_PA_1  ACCACTCCCTAATCTCAAGTACCCAGGGACACAAACACTGCGGAAGGCCGCAGGGACCTCTGCCTAGGAAAACCAGAGACCTTTGTTCACGTGTTTATCTGCTGACCTTC
SVA_PA_2  .........................................................................................T....................
SVA_PA_3  .........................................A...............................................T....................
SVA_PA_4  .........................................................................................T....................
SVA_PA_5  ............................G............A...............................................T..........C.........
SVA_PA_6  .........................................A...............................................T..........C.........
SVA_PA_7  ......................T.....G............A.............T.................................T..........C.........
SVA_PA_8  ............................G..G.........A.............T............................A....T..........C.........
SVA_PA_9  ............................G............A.............T.................................T..........C.........
SVA_PA_10 ................C...........G............A.............T.................................T..........C.........
SVA_PA_11 ............................G............A.............T.................................T..........C.........
SVA_PA_12 .........................................................................................T....................

SVA_PA_1  TCTCCACTATTATCCTATGACCCTGCCACATCCCCCTCTCCGAGAAACACCCAAGAATGATCAA
SVA_PA_2  ............................T...................................
SVA_PA_3  ............................T...................................
SVA_PA_4  ............................T...................................
SVA_PA_5  ............................T...................................
SVA_PA_6  ..........C.................T...................................
SVA_PA_7  ............................T...................................
SVA_PA_8  ........................C...T...................................
SVA_PA_9  ........................C...T.............................C.....
SVA_PA_10 ........................C...T.............................C.....
SVA_PA_11 ..........C.............C...T.............................C.....
SVA_PA_12 ............................T...................................
```

B

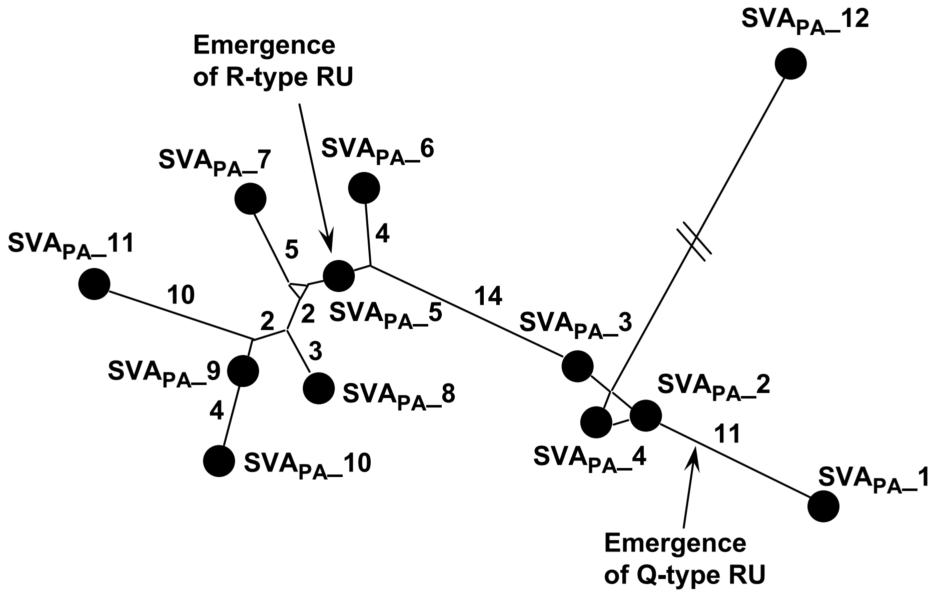

SVA$_{PA}$_12
Emergence
of R-type RU
SVA$_{PA}$_7
SVA$_{PA}$_6
SVA$_{PA}$_11
5
4
10
2
2
14
SVA$_{PA}$_5
SVA$_{PA}$_3
3
SVA$_{PA}$_9
SVA$_{PA}$_8
SVA$_{PA}$_2
4
11
SVA$_{PA}$_4
SVA$_{PA}$_10
SVA$_{PA}$_1
Emergence
of Q-type RU

Figure S4 Multiple alignment of the consensus sequences of the LAVA subfamilies' 3' parts (U1-*Alu*Sz-U2-L1ME). Subfamilies were obtained by manual sorting. The alignment shown was used for generation of the network presented in figure 5. *M. mulatta* and *N. leucogenys* represent the source sequence of the LAVA 3' part in *Hydroxysteroid (17-beta) dehydrogenase 3* (*HSD17B3*) intron 2.

```
M.mulatta     CTGCCATGGAGGCCAGACGCAGTACAGGGGCTGGACGTGGTGGCTCACACCTGTAATCCCAGCACTCTGGGAAGCTGAGGCGGGTTGATCACTTGAGGTTAGGAGTTTGAGACCAGCCTGACCAACACGGCAAAACCCCG
N.leucogenys  ..A...C................GTG.....C....A...................................G..CA....A...........A....C........C............G......T...G......T.
LAVA_A1       ..A...C................GTG......................G......G................G..C......................C........C............G......T...G....----
LAVA_A2       ..A...C................GTG......................G......G................G..C......................C........C.........T..G......T...G....----
LAVA_B1A      ..A...C..............A.GTG......................G......G................G..C......................C........C............G......T...G....----
LAVA_B1D      ..A...C..............A.GTG......................G......G............A...G..C....A.................C........C............G......T...G....----
LAVA_B1R      ..A...C..............A.GTG......................G......G................G..C......................C........C............G......T...G....----
LAVA_B1B      ..A...C..............A.GTG......................G.....GG................G..C......................C........C..C.........G......T...G....----
LAVA_B1M      ..A...C..........A...A.GTG......................G......G................G..C......................C........C............G......T...G....----
LAVA_B2R2     ..A...CA.........A...A.GTG......................G......G......T.........G..C......................C........C............G......T...G....----
LAVA_B2R1     ..A...CA.........A...A.GTG......................G......G......T.........G..C......................C........C............G......T...G....----
LAVA_B2A      ..A...CA.........A...A.GTG.........T............G......G......T.........G.........................C......C.C............G......T...G....----
LAVA_B2C      ..A...CA.........A...A.GTG......................G......G......T.........G..C......................C........C............G......T...G....----
LAVA_B2B      ..A...CA.........A...A.GTG..........A...........G......G......T.......A.G..C......................C........C............G......T...G....----
LAVA_B2E      ..A...CA.........A...A.GTG......................G......G......T.........G..C......................C........C............G......T..TG....----
LAVA_B1G      ..A...C..........A...A.GTG......................G......G................G..C......................C........C............G......T...G....----
LAVA_B1F      ..A...C..........A...A.GTG......................G......G....G..G........G..C..................C...C........C............G......T...G....----
LAVA_B1L      ..A...C..........A...A.GTG......................G......G................G..C..................C...C........C............G......T...G....----
LAVA_C1       ..A...C..........A...A.GTG......................G.....GG...............GG..C..................C...C........C........T...G......T...G....----
LAVA_C2       ..A...C..........A...A.GTG......................G.....GG....G..........GG..C..................C...C........C........T...G......T...G....----
LAVA_C3       ..A...C..........A...A.GTG...................T..G.....GG...............GG..C.........C............C........C............G......T...G....----
LAVA_C4       ..A...C..........A...A.GTG......................G.....GG....G..........GG..C..................C...C........C........T...G......T...G....----
LAVA_D        A.A...C..........A...A.CTG......................G.....GG....G..........GG..C..................CG..C........C........T...G......T...G....----
LAVA_E        ..A...C..........A...A.GTG......................G.....GG....G..........GG..-..................CG..C........C........T...G.....TT...G....----
LAVA_F        ..A..............A...A.GTG......................G.....GG....G..........GG..-..................CG..C........C........T...G.....TT...G....----
LAVA_F0       ..A..............A...A.GTG......................G.....GG....G..........GG..-..................CG..C........C........T...G.....TT...G....----

M.mulatta     TCTCTACTAAAAATACAACAAACAGACAAA--AA--AACCCAGCAATAATAAAACAGGTCTACCCTGGCAGTCATACTCTAATTTTTTCTATTTGCCTCCCTTTCTGATCCTTTATCCCACTTTCCTTTTCTTTCTCCTC
N.leucogenys  ........................A..C..CC..CC..........C.....................-.........................T........................G.....T.......C...T..
LAVA_A1       ---A..TG...............CA..C..CC..CC..........C..C..................-.........................T..............................T.......C...T..
LAVA_A2       ---A..TG................A..C..CC..CC..........C..C..................-.........................T..............................T.......C...T..
LAVA_B1A      ---A..TG............G...A..C..CC..CC..........C..C..................-.........................T..............................T.......C...T..
LAVA_B1D      ---A..TG.......T....G...A..C..CC..CC..........C..C..................-.........................T..............................T.......C...T..
LAVA_B1R      ---A..TG............G...A..C..CC..CC........G.C..C..................-.........................T..............................T.......C...T..
LAVA_B1B      ---A..TG............G...A..C..CC..CC.......TG.C..C..................-.........................T..............................T.......C...T..
LAVA_B1M      ---A..TG............G...A..C..CC..CC........G.C..C..................-.........................T..............................T.......C...T..
LAVA_B2R2     ---A..TG............G...A..C..CC..CC........G.C..C..................-.........................T..............................T.......C...T..
LAVA_B2R1     ---A..TG...........TG...A..C..CC..CC....A...G.C..C..................-.........................T..............................T.......C...T..
LAVA_B2A      ---A..TG...........TGT..A..C..CC..CG....A...G.C..C..................-.........................T..............................T.......C...T..
LAVA_B2C      ---A..TG...........TG...A..C..CC..CC....A...G.C..C..................-....G....................T..............................T.......C...T..
LAVA_B2B      ---A..TG...........TG...A..C..CC..CC....A...G.C..C..................-.........................T..............................T.......C...T..
LAVA_B2E      ---A..TG............G...A..C..CC..CC....A...G.C..C..................-.........................T..............................T.......C...T..
LAVA_B1G      ---A..TG............G...A..C..CC..CC........G.C..C..................-.........................T..............................T.......C...T..
LAVA_B1F      ---A..TG............G...A..C..CC..CC........G.C..C..................-.........................T..............................T.......C...T..
LAVA_B1L      ---A..TG............G...A..C..CC..CC........G.C..C..................-.........................T..............................T.......C...T..
LAVA_C1       ---A..TG............G...A..C..CC..CC.......TG.C..C..................-.........................T..............................T.......C...T..
LAVA_C2       -----.TG............G...A..C..CC..CC...T...TG.C..C..................-.........................T..............................T.......C...T..
LAVA_C3       ---A..TG............G...A..C..CC..CC........G.C..C..................-.........................T..............................T.......C...T..
LAVA_C4       ---A..TG............G...A..C..CC..CC...T...TG.C..C..................-.........................T..............................T.......C...T..
LAVA_D        ---A..TG............G...A..C..CC..CC...T...TG.C..C............T.....-.........................T..............................T.......C...T..
LAVA_E        -----.TG............G...A..C..CC..CC...T...TG.C..C..................-.........................T..............................T.......C...T..
LAVA_F        -----.TG............G...A..C..CC..CC...T...TG.C..C..................-.........................T..............................T.......C...T..
LAVA_F0       -----.TG............G...A..C..CC..CC...T...TG.C..C..................-.........................T...................G..........T.......C...T..
```

```
M.mulatta      CTTCTCCTCCTTCTTTGTCAAAGACATAGAGGATTGAGTTATTATCATTGATCCATACACAGTCCCTCTCTCATTTATTTTCTTTCATTCCCACCCCCT-ATTTCTATTCCCCGTCTTCCCATATGCAACCTTCCTAATA
N.leucogenys   ........T..................................................A.......T..............................CC.........T.............G................
LAVA_A1        ........T..................................................A......................................C-.......................G................
LAVA_A2        ........T..................................................A......................................C-............G..........G................
LAVA_B1A       ........T.............----.................................A.........................A............C-.......................G................
LAVA_B1D       ........T.............----.................................A.........................A.....T......C-.......................G................
LAVA_B1R       ........T.............----.................................A.........................A............C-.......................G................
LAVA_B1B       ........T.............----.................................A.........................A............C-.......................G................
LAVA_B1M       ........T.............----.................................A.........................A............C-.......................G................
LAVA_B2R2      ........T.............----.................................A.........................A............C-.......................G................
LAVA_B2R1      ........T.............----.................................A.........................A............C-.......................G................
LAVA_B2A       ........T.............----.................................A.........................A............C-.......................G................
LAVA_B2C       ........T.............----.................................A.........................A............C-.......................GG...............
LAVA_B2B       ........T...T.........----.................................A.........................A............C-..............C........G................
LAVA_B2E       ........T.............----.................................A.........................A............C-.......................G................
LAVA_B1G       ........T.............----.................................A.........................A........T...C-.......................G................
LAVA_B1F       ........T.............----.................................A...............C.........A........T...C-.......................G................
LAVA_B1L       ........T.............----.................................A.........................A........T...C-.......................G................
LAVA_C1        ........T.............----.....................C.........T.A.........................A........T...C-.......................G................
LAVA_C2        ........T.............----.....................C.........T.A.....................----A............C-..............A........G................
LAVA_C3        ........T.............----.....................C.........T.A.....................----A............C-..............A........G................
LAVA_C4        ........T.............----.....................C.........T.A.....................----A............C-..............A........G................
LAVA_D         ........T....---......----.....................C.........T.A.....................----G........T...C-.......................G................
LAVA_E         .......CT.............----.....................C.........T.A.....................----A.C..........C-..............A........G..T.............
LAVA_F         .......CT.............----.....................C.......C.T.A...........ACC.......----A.C..........C-..............A........G..T.............
LAVA_F0        .......CT.............----.....................C.......C.T.A...........ACC.......----A.C..........C-..............A..G.....G..T.............

M.mulatta      TGTTTGATGTGCATCTTTTTGTTTGTATGTACTTTTAGAAAATGTTTATTGTTTT-GTGTGC
N.leucogenys   ........A......................T................C......-......
LAVA_A1        ........A......................T.......................-......
LAVA_A2        ........A......................T.......................-......
LAVA_B1A       ........A......................T.......................-......
LAVA_B1D       ........A......................T.......................-......
LAVA_B1R       ........A......................T.......................-......
LAVA_B1B       ........A......................T.......................-......
LAVA_B1M       ........A......................T.......................-......
LAVA_B2R2      ........A..............................................-......
LAVA_B2R1      ........A..............................................-......
LAVA_B2A       ........A..............C...............................-......
LAVA_B2C       ........A..............................................-......
LAVA_B2B       ........A..............................................-......
LAVA_B2E       ........A.A............................................-......
LAVA_B1G       ........A......................T.......................-......
LAVA_B1F       ........AC.....................T.......................-......
LAVA_B1L       ........AC.....................T.......................-......
LAVA_C1        ........AC.....................T.......................-......
LAVA_C2        ........AC.....................T.......................-......
LAVA_C3        ........AC.....................T.......................T......
LAVA_C4        ........AC.....................T.......................T......
LAVA_D         ........AC.....................T.......................T......
LAVA_E         ........AC.....................T.......................T......
LAVA_F         ........AC.....................T.......................T......
LAVA_F0        ........AC.....................T.......................T......
```

Figure S5 Alignments of the VNTR regions of orthologous SVA_D elements in human (HSA) and chimpanzee (PTR). Microhomologies flanking breakpoints are highlighted in yellow. Repeat unit (RU) types are annotated on top of the sequences. Variant RUs in chimpanzee are indicated in red below the respective RU. Changes in the repeat unit patterns are given below the respective alignment.

```
                          A                                         B
D11_HSA  CCTCTGCCTGGCCGCCACCCCATCTGGGAAGTGAGGAGCG-TCTCTGCCTGGCCGCCCATCGTCTGGGATGTGAGGAGCC-
D11_PTR  CCTCTGCCCGGCCGCCACCCTGTCTGCGAAGTGAGGAGCA-TCTCTGCCTGGCCGCCCATCGTCTGGGATGTGAGGAGCC-

                          C                                         A
D11_HSA  CCTCTGCCTGGCTGCCCAGTCTGGAAAGTGAGGAGCG-TCTCTGCCCGGCCGCCATCCCATCTAGGAAGTGAGGAGCG-
D11_PTR  CCTCTGCCTGGCTGCCCAGTCTGGAAAGTGAGGAGCG-TCTCTGCCTGGCTGCCATCCCATCTAGGAAGTGAGGAGCG-

                          A                                         B′
D11_HSA  CCTCTTCCCAGCCGCCATCCCATCGAGGAAGTGAGGAGCG-TCTCTGCCCGACCACCCATCGTCTGAGATGTGAGGAGCG-
D11_PTR  CCTCTTCCCAGCCGCCATCCCATCGAGGAAGTGAGGAGCG-TCTCTGCCCAGCCACCCATCATCTGAGATGTGAGGAGCG-

                          C                                         A
D11_HSA  CCTCTGCCCCGCCGCCCCGTCTGGGATGTGAGGAGCG-CCTCTGCCTGGCCGCGACCCCGTCTGGGAGGTGAGGAGCG-
D11_PTR  CCTCTGCCCTGCCGCCCCGTCTGGGATGTGAGGAGCG-CCTCTGCC---------------------------------

                          C                                         A
D11_HSA  TCTCTGCCCGGCCGCCCCGTCTGAGAAGTGAGGAGAC-CCTCTGCCCGGCAGCCGCCCCGTCTGGGAAGTGAGGAGCG-
D11_PTR  --------CGGCCGCCCCGTCTGAGAAGTGAGGAGAC-CCTCTGCCCGGCAGCCGCCCCGTCTGGGAAGTGAGGAGCA-

                          F                                                K
D11_HSA  TCTCCGCCCGGCAGCCACCCCGTCCGGGAGGGAGGTGGGGGTCAGC-CCCCCGCCCGGCCAGCCGCCCCGTCCGGGAGGGAGGTGCGGGTCAGC-
D11_PTR  TCTCCGCCCAGCAGCCACCCCGTCCGGGAGGGAGGTGGGGG------------------------------------------------------

                          K                                                 K
D11_HSA  CCCCCGCCTGGCCAGCCGCCCCGTCCGGGAGGGAGGCGGGGGTCAGC-CCCCCGCCCGGCCAGCCGCCCCGTCCAGGAGGGAGGTGGGGGTCAGC-
D11_PTR  -----------------------------------------------------------------------------------------GTCAGC-

                          K                                                 K
D11_HSA  CCCCCGCCCGGCCAGCCGCCCCGTCCGGGAGGGAGGTGGGGGTCAGC-CCCCTGCCCGGCCAGCCGCCCCGTCCGGGAGGGAGGTGGGGGTCAGC-
D11_PTR  CCCCCGCCCGGCCAGCCGCCCCGTCCGGGAGGGAGGTGG----------------------------------------------GGGGGTCAGC-

                          G                                         C′
D11_HSA  CCCCCGCCCGGCCAGCCGCCCCGTCCAGAAGGTGAGGGGCG-CCTCTGCCCGGCCGCCCCTACTGGGAAGTGAGGAGCC-
D11_PTR  CCCCCACCCGGCCAGCCGCCCCGTCCGGA-GGTGAGGGGCG-CCTCTGCCCGGCCGCCCCTACTGGGAAGTGAGGAGCC-

                          T
D11_HSA  CCTCTGCCTGGCCACCACCCCGTCTGGGAGGTG
D11_PTR  CCTCTGCCCGGCCACCACCCCGTCTGGGAGGTG
```

CAC ↔ C-C

FKKKKKG ↔ F---K-G

Figure S5 – continued

```
                             A                                         B
D12_HSA    CCTCTGCCCGGCCACCACCCCGTCTGGGAAGTGAGGAGCG-TCTCTGCCTGGCCGACCATCGTCTGGGATGTGAGGAGCC-
D12_PTR    CCTCTGCCCGGCCACCACCCCGTCTGGGAAGTGAGGAGCG-TCTCTGCCTGGCCGACCATCGTCTGGGATGTGAGGAGCC-

                             C                                         A
D12_HSA    CCTCTGCCTGGCTGCCCAGTCTGGAAAGTGAGGAGCG-TCTCTGCCCGGCCGCCATCCCATCTAGGAAGTGAGGAGCG-
D12_PTR    CCTCTGCCTGGCTGCCCAGTCTGGAAAGTGAGGAGCG-TCTCTGCCTGGCCGCCATCCCATCTAGGAAGTGAGGAGCG-

                             B′                                        C
D12_HSA    TCTCTGCCCGGCCACCCATCGTCTGAGATGTGGGGAGCG-CCTCTGCCCCGCCGCCCCGTCTGGAATGTGAGGAGCG-
D12_PTR    TCTCTGCCCGGCCGCCCATCGTCTGAGATGTGGGGAGCG-CCTCTGCCCCGCCACCCCGTCTGGAATGTGAGGAGCG-

                             A                                         C
D12_HSA    CCTCTGCCCGGCCGCAACCCTGTCTGGGAGGTGAGGAGCG-TCTCTGCCCGGCCGCCCCGTCTGAGAAGTGAGGAGAC-
D12_PTR    CCTCTGCCCGGCCGCCACCCCGTCTGGGAGGTGAGGAGCG-TCTCTGCCCGGCCGCCCCGTCTGGGAAGTGAGGAGAC-

                             A                                         A
D12_HSA    CCTCTGCCTGGCAACCGCCCCGTCTGAGAAGTGAGGAACC-CCTCCACCCGGCAGCCACCCCGTCTGGGAAGTGAGGAGCG-
D12_PTR    CCTCTGCCTGGCAACCGCCCCGTCTGAGAAGTGAGGAGCC-CCTCCGCCCGGCAGCCACCCCGTCTGGGAAGTGAGGAGCG-

                             ?
D12_HSA    TCTCCGCCCGGCAGCCACCCCACCCGGGAGGGAGGTG-----------------------------------------------------------------
D12_PTR    TCTCCGCCCGGCAGCCGCCCCGTCCGGGAGGGAGGTGGGGGTCAG-CCCCCGCCAGGCCAGCCGCCCCATCCGGGAGGGAGGTGGGGGGGTCAGC-
                             ?                                                       K
                                                                       C′
D12_HSA    ----------------------------------AGGGGTG-CCTCTGCCCGGCCGCCCCTACTGGGAAGTGAGGCGCC-
D12_PTR    CCCCCGCCCGGCCAGCCGCCCCGTCCGGGAGGTGAGGGGCG-CCTCTGCCCGGCCGCCCCTACTGGGAAGTGAGGAGCC-
                             G
                             T
D12_HSA    CCTCTGCCTGGCCACCACCCCGTCTGGGAGGTG
D12_PTR    CCTCTGCCCGACCACCACCCCGTCTGGGAGGTG
```

A?--C' ↔ A?KGC′

```
                             A                                         B
D13_HSA    CCTCTGCCCGGCGGCCACGCCGTCTGGGAAGTGAGGAGCG-TCTCTGCCCGGCCGCCCATCATCTGGGATGTGAGGAGCC-
D13_PTR    CCTCTGCATGGCCGCCACCCCGTCTGGGAAGTGAGGAGCG-TCTCTGCCTGGCCGCCCATCGTCTGGGACGTGAGGAGCC-

                             C                                         A
D13_HSA    CCTCTGCCTGGCTGCCCAGTCTGGAAAGTGAGGAGCG-TCTCTGCCCGGCCGCGATCCCATCTAGGAAGTGAGGAGCG-
D13_PTR    CCTCTGCCTGGCTGCCCAGTCTGGAAAGTGAGGAGCG-TCTCTGCCCGGCC----------------------------

                             A                                         B′
D13_HSA    CCTCTTCCCGGCTGCCATCCCATCTAGGAAGTGAGGAGCG-TCTCTGCCCGGCCGCCCATCGTCTGATATGTGGGGAGCG-
D13_PTR    -------------GCCATCCCACCTGGGAAGTGAGGAGCG-TCTCTGCCCGGCCGCCCATCGTCTGAGATGTGGGGAGCG-

                                                                       C
D13_HSA    CCTCTGCCC-----------------------------------------CGCCGCCCCGTCTGGGATGTGAGGAGCG-
D13_PTR    CCTCTGCCCGGCCGCGACCCCATCTGGGATGTGAGGAGCA-TCTCTGCCCAGCCGCCCCGTCTGAGAAGTGAGGAGAC-
                             A                                         C
                             C                                         A
D13_HSA    CCTCTGCCCA---GCCGCCCCGTCTGAGAAGTGAAGAG-CCCCTCCGCCCGGCAGCCGCCCCATCTGGGAAGTGAGGAGCG-
D13_PTR    CCTCCGCCCAGCAGCCGCCCCGTCTGAGAAGTGAGGAG-CCCCTCTGCCCAGCAGCTGCCCCGTCTGGGAAGTGAGGAGCG-
                             A                                         A
                             F                                                G
D13_HSA    TCTCCGCCCGGCAGCCAACCCGTCTGGGAGGGAGGTGGGGGGTCAG-CCCCCACGCGGCCGGCCGCCCCGTCCGAGAAGTGGGGGGCG-
D13_PTR    TCTCCGCCCGGCAGCCACCCCACCCGGGAGGGAGGTGGGGG-TCAG-CCCCCGCCCGGCCAGCCACCCCATCTGGGAGGTGGGGGGCG-

                             C′                                  T
D13_HSA    CCTCTGCCCGGCCGCCCCTACTGGGAAGTGAGGAGCC-CCTCTGCCCGGCCACCACCCCGTCTGGGAGGTG
D13_PTR    CCTCTGCCCGGCCGCCCCTACTGGGAAGTGAGGAGCC-CCTCTGCCCGGCCACCACCCCATCTGGGAGGTG
```

CAAB′ ↔ C-AB′

B′-C ↔ B′AC

C ↔ A

Figure S5 – continued

```
                           A                                        B
D14_HSA CCTCTGCCTGGCCGCCATCCCGTCTGGGAAGTGAGGAGCG-TCTCTGCCTGGCCGCCCATCGTCTGGGATGTGAGGAGCC-
D14_PTR CCTCTGCCCGGCCGCCACCCCGTCTGGGAAGTGAGGAGCG-TCTCTGCCTGGCCGCCCATCGTCTGGGATGTGAGGAGCC-

                           C                                        A
D14_HSA CCTCTGCCTGGCTGCCCAGTCTGGAAAGTGAGGAGCG-TCTCTGCCCGGCCGCCATCCC--------------------
D14_PTR CCTCTGCCTGGCTGCCCAGTCTGGAAAGTGAGGAGCG-TCTCTGCCCGGCCGCCATCCCATCTAGGAAGTGAGAAGCG-

                                                                    B′
D14_HSA ---------------------ATCTAGGAAGTGAGGAGCG-TCTCTGCCCGGCCGCCCATCGTCTGA--------------
D14_PTR CCTCTTCCTGGCCGCCATCACATCTAGGAAGTGAGGAGCG-TCTCTGCCCGGCCGCCCATCGTCTGAGATGTGGGGAGCG-
                           A                                        B′
                                                                    A
D14_HSA ------------------------GATGTGAGGAGCG-CCTCTGCCCGGCCGCGACCCCGTCTGGGAGGTGAAGAGCG-
D14_PTR CCTCTGCCCCGCCGCCCCGTCTGGGATGTGAGGAGCG-CCTCTGCCCGGCCGTGACCCCGTCTGGGAGGTGAAGAGCA-
                           C                                        A
                           C                                        A
D14_HSA TCTCTGCCCAGCCGCCCCGTCTGAGAAGTGAGGAGAC-CCTCTGCCTGGCAACCACCCCGTCTGAGAACTGAGGAGCC-
D14_PTR TCTCTGCCCAGCCGCCCCGTCTGAGAAGTGAGGAGAC-CCTCTGCCTGGCAACCACCCCATCTGAGAACTGAGGAGCC-

                           A                                        A
D14_HSA CCTCTGCCCGGCAGCCGCCCCGTCTGAGAAGTGAGGAGCC-TCTCCGCCCGGCAACCACCCCGTCTGGGAAGTGAGGAGCC-
D14_PTR CCTCCGCCCGGCAGCCGCCCCGTCTGAGAAGTGAGGAGCC-TCTCCGCCCGGCAACCACCCCGTCCGGGAAGTGAGGAGTG-

                                                                                   G
D14_HSA CCTCTG----------------------------------------------------CCCGGCCAGCCGCCCCGTCCGGGAGGTGAGGGGCG-
D14_PTR TCTCCGCCCGGCAGCCACCCCGTCCGGGAGGGAGGTGGGGGGGGGGTCAGC-CCCCCGCCCGGCCAGCCGCCCCGTCCGGGAGGTGAGGGGCG-
                           F                                                       G
                           C′                                       K
D14_HSA CCTCTGCCCGGCCGCCCCTACTGGGAAGTGAGGAGCC-CCTCTGCCCAGCCAGCCGCCCCGTCCAGGAGGGAGGTGGGGGGGTCAGC-
D14_PTR CCTCTGCCCGGCCGGCCCTACTGGGAAGTGAGGAGCC-CCTCTGCCCGGCCAGCCACCCTGTCCAGGAGGGAGGTGGGGGGGTCAGC-

                           K                                                       K
D14_HSA CCCCTGCCCGGCCAGCCGCCCCGTCCGGGAGGGAGGTGGGGGGGTCAGC-CCCCCGCCCAGCCAGCCGCCCCGTCCGGGAGGGAGGTGG-----------
D14_PTR CCACCGTCCAGCCAGCCGCCCCGTCCGGGAGGGAGGTGGGGGGGTCAGC-CCCCCGTCCGGCCAGCCGCCCCGTCCGGGAGGGAGGTGGGGGGGTCAGC-

                                                                                   G
D14_HSA -------------------------------------GGGGGGGGTCAGC-CCCCCGCCCGGCCAGCCGCCCCGTCCGGGAGGTGAGGGGTG-
D14_PTR CCCACGCCCGGCCAGCTGCCCCGTCCGGGAGGGAGGTGGGGGGGGTCAG--CCCCCGCCCGGCCAGCCGCCCCGTCCGGGAGGTGAGGGGCG-
                           K                                                       G
                           C′                                 T
D14_HSA CCTCTGCCCGGCCACCCCTACTGGGAAGTGAGGAGCC-CCTCTGCCTGGCCACCACCCCGTCTGGGAGGTG
D14_PTR CCTCTGCCCGGCCACCCCTACTGGGAAGTGAGGAGCC-CCTCTGCCTGGCCACCACCCCGTCTGGGAGGTG
```

CA-B′ ↔ CAAB′

B′-A ↔ B′CA

A-G ↔ AFG

K-G ↔ KKG

Figure S5 – continued

```
                          A                                         B
D15_HSA CCTCTGCCCGGCCGCCGCCCTGTCTGGGAAGTGAGGGGCG-TCTCTGCCTGGCCGCCCATCGTCTGGGATGTGAGGAGCC-
D15_PTR CCTCTGCCCGGCCGCCGCCCTGTCTGGGAAGTGAGGGGCG-TCTCTGCCT-------------------------------

                          C                                         A
D15_HSA CCTCTGCCTGGCTGCCCAGTCTGGAAAGTGAGGAGCG-TCTCTGCCCGGCCACCATCCTGTCTAGGAAGTGAGAAGCG-
D15_PTR -------------------------------------------------------------------------------

                          B                                         C
D15_HSA TCTCTGCCTGGCCGCCCATTGTCTGGGATGTGAGGAGCC-CCTCTGCCTGGCTGCCCAGTCTGGAAAGTGAGGAGCG-
D15_PTR ------------------------------------------------------------------------------

                          A                                         A
D15_HSA TCTCTGCCCGGCCGCCATCCCATCTAGGAAGTGACGAGCG-TCTCTTCCCGGCCGCCATCCCATCTAGGAAGTGAAGAGCG-
D15_PTR ---------GGCCGCCATCCCATCTAGGAAGTGACGAGCG-TCTCTTCCCGGCCGCCATCCCATCTAGGAAGTGAGGCGCG-

                         B′                                         C
D15_HSA TCTCTGCCCGGCTGCCCATCGTCTGAGATGTGGGGAGAG-CCTCTGCCCCGCCGCCCCGTCTGGGATGTGAGGAGCG-
D15_PTR TCTCTGCCCGGCCGCCCATTGTCTGAGATGTGGGGAGAG-CCTCTGCCCCGCCACCCCGTCTGGGATGTGAGGAGCG-

                         A                                          C
D15_HSA CCTCTGCCCGGCCGCGACCCCGTCTGGGAGGTGAGGAGCG-TCTCTGCCTGGCCGCCCC--------------------
D15_PTR CCTCTGCCCGGCCGCGACCCCGTCTGGGAGGTGAGGAGCG-TCTCTGCCTGGCCGCCCCGTCTGAGAAGTGAGGAGAC-

                                                                    F
D15_HSA ---------------------GTCTGAGAAGTGAGGAGCC-CCTCCACCCGGCAGCCGCCCCGCCCGGGAGGGGGGTGGGGGGGGGTCAGC-
D15_PTR CCTCCGCCCAGCAGCCGCCTCGTCTGAGAAGTGAGGAGCC-CCTCCGCCCGGCAGCCGCCCCACCTGGGAGTGGGGTGGGGGGG--TCAGC-
                         A                                          F
                                                                                           K
D15_HSA CCCCCGCCCGGCCAGCCA----------------------------------------------------CCCTGTCTGGGAGGGAGGTGTGGGGGGGGTCAGC-
D15_PTR CCCCCGCCCGGCCAGCCGCCCCGTCCGGGAGGGAGGTGTGGGGGAGTCAGCCCCCCCGCCCGGCCAGCCGCCCCGTCCAGGAGGGAGGTGTGGGGGGGGTCATC-
                         K                                                                 K
                         K                                          G(Δ)
D15_HSA CCCCTGCCCGGCCAGCCGCCCCGTCCGGGAGGGAGGTGTGGGGGGGTCAGC-CGTCCCGTCCGGGAGGTGAGGGGCG-
D15_PTR CCCCCGCCCGGCCAGC------------------------------------CGTCCCATCCGGGAGGTGAGGGGCG-
                      K(Δ)                                          G(Δ)
                          C′                                 T
D15_HSA CCTCTGCCCGGCCGCCCCTACTGGGAAGTGAGGAGC-CCCTCTGCCCAGCCACCACCCCGTCTGGGAGGTG
D15_PTR CCTCTGCCCGGCCGCCCCTACTGGGAAGTGAGGAGC-CCCTCTGCCCGGCCACCACCCCGTCTGGGAGGTG
```

ABCABCA ↔ A-----A

AC-F ↔ ACAF

F-KKG(Δ)C′ ↔ FKKK(Δ)G(Δ)C′

Figure S5 – continued

```
                            A                                         B
D16_HSA   TCTCCGCCCGGCTGCCACCCCATCTGGGAAGTGAGGAGCA-TCTCTGCCTGGCCGCCCATCGTCTGGGATGTGAGGAGCC-
D16_PTR   CCTCCACCCGGCTGCCACCCCATCTGGGAAGTGAGGAGTG-TCTCTGCCTGGCCGCCCATCATCTGGGATGTGAGGAGCC-

                            C                                         A
D16_HSA   CCTCTGCCTGGCTGCCCAGTCTGGGAAGTGAGGAGCG-CCTCTTCCCGGCCGCCATCCCATCTAGGAAGTGAGGAGCG-
D16_PTR   CCTCTGCCTGGCTGCCCAGTCTGGGAAGTGAGGAGCG-CCTCTTCCCGGCCGCCATCCCATCTAGGAAGTGAGGAGCA-

                            B'                                        C
D16_HSA   TCTCTGCCCAGCCGCCCATCGTCTGAGATGTGGGGAGCG-CCTCTGCCCCGCCGCCCCGTCTGGGATGTGAGGAGCG-
D16_PTR   TCTCTGCCCA----------------------------------------GCCGCCCCATCTGGGATGTGAGGAGCA-
                                                                      C
                            A                                         C
D16_HSA   CCTCTGCCCGGCCGCGACCCTGTCTGGGAGGTGAGGAGCG-TCTCTGCCTGGCCACCCTGTCTGAGAAGTGAGGAGCC-
D16_PTR   CCTCTGCCCGGCCGCGACCCCGTCTGGGAGGTGAGGAGCG-TCTCTGCCCGGCCACCCTGTCTGAGAAGTGAGGAGCC-

                            A
D16_HSA   CCTCCGCCCGGCAGCCACCCCATCTGAGAAGTG-----------------------------------------AGGAGCA-
D16_PTR   CCTCCACCCGGCAGCCACCCCGTCTGAGAAGTGAGGAGCC-CCTCCGCCCGGCAGCCATCCCATCTGGGAAGTAAGGAGCA-
                            A                                         A
                            F                                                K
D16_HSA   TCTCCGCCCGGCAGCCACCCCGTCCGGGAGGGAGGTGGGGGTCAG-CCCCCACCCGGCCAGCCGCCCCGTCCGGGAGGGAGGTGGGGGGTCAG-
D16_PTR   TCTCCACCTGGCAGCCACCCCGTCCGGGAGGGAGGTGGGGGTCAG-CCCCCACCCAGCCAGCCGCCCCGTCCGGGAGGGAGGTGGGGGGTCAG-

                            G                                         C'
D16_HSA   CCTCCGCCCGGCCAGCCGCCCCGTCCGGGAGGTGGGGGGCG-CCTCTGCCCGGCCGCCCCTTCTGGGAAGTGAGGAGCC-
D16_PTR   CCTCCGCCCGGCCAGCCACCCCGTCCGGGAGGTGGGGGGCG-CCTCTGCCTGGCGGCCCCTTCTGGGAAGTGAGGAGCC-

                            T
D16_HSA   CCTCTGCCCGGCCACCACCCCGTCTGGGAGGTG
D16_PTR   CCTCTGCCCGGCCACCACCCCGTCTGGGAGGTG
```

AB′C ↔ A-C

A-F ↔ AAF

```
                            A                                         A
D17_HSA   CCTCTGCCTGGCCGCCATCCCATCTAGGAAGTGAGGAGCA-CCTCTTCCCGGCCGCCATCCCATCTAGGAAGTGAGGAGCG-
D17_PTR   CCTCTGCCTGGCCGCCATCCCATCTAGGAAGTGAGGAGCA-CCTCTTCCCGGCCGCCATCCCATCTAGGAAGTGAGGAGCG-

                            B'                                        C
D17_HSA   TCTCTGCCCAGCCGCCCATCGTCTGAGATGTGGGGAGCG-CCTCTGCCCCGCTGCCCCGTCTGGGATGTGAGGAGCG-
D17_PTR   TCTCTGCCCAACCGCCCATCGTCTGAGATGTGGGGAGCG-CCTCTGCCCCACTGTCCTGTCTGGGATGTGAGGAGCG-

                            A                                         C
D17_HSA   CCTCTGCCCGGCCACAACCCCGTCTGGGAGGTGAGGAGCA-TCTCTGCCCGGCCGCCCCATCTGAGAAGTGAGGAGAC-
D17_PTR   CCTCTGCCCGGCCACAACCCCGTCTGGGAGGTGAGGAGCG-TCTCTGCCCGGCTGCCCCATCTGAGAAGTGAGGAGAC-


D17_HSA   CCTCTGCCC-------------------------------------------------------------------------
D17_PTR   CCTCCGCCCAGCAGCCGCCCCGTCTGAGAAGTGAGGAGAC-CCTCCGCCCGGCAGCCGCCCCGTCTGAGAAGTGAGGAGAC-
                            A                                         A
                                                                      A
D17_HSA   --------------------------------------------------GGCAGCTGCCCCGTCTGAGAAGTGAGGAGCC-
D17_PTR   CCTCCGCCCGGCAGCCGCCCCGTCTGAGAAGTGAGGAGAC-CCTCCGCCTGGCAGCTGCCCCATCTGAGAAGTGAGGAGCC-
                            A                                         A
                            A                                         F
D17_HSA   CCTCTGCCCGGCAGCCACCCCGTCTGGGAAGTGAGGAGCG-TCTCCGCCCAGCAGCCACCCCGTCCGGGAGGGAGGTGGGGGTCAG-
D17_PTR   CCTCTGCCCGGCAGCCACCCCGTCTGGGAAGTGAGGAGCG-TCTCCGCCCGGCAGCCACCCCGTCCGGGAGG-AGGTGGGGGTCAG-

                            G
D17_HSA   CCCCCGCCTGGCCAGCTGCCCCATCTGGGAGGT--------------------------------------------------------------
D17_PTR   CCCCCGCCTGGCCAGCCGCCCCATCTGGGAGGGAGGTGGGGGTCAG-TCCCCACCCCGGCCAGCCGCCCCGTCCGGGAGGGAGGTGGGGGTCAG-
                            K                                                        K
                                                                      C'
D17_HSA   ----------------------------------GGGGGGCG-CCTCTGCCCGGCCGCCCCTTCTGGGAAGTGAGGAGCC-
D17_PTR   CACCCGCCCGGCCAGCCGCCCCGTCTGGGTGGTGGGGGGGCG-CCTCTGTCCGGCCGCCCCTTCTGGGAAGTGAGGAGCC-
                            G                                         C'
                            T
D17_HSA   CCTCTGCCCAGCCACCACCCCGTCTGGGAGGTG
D17_PTR   CCTCTGCCCGGCCACCACCCTGTCTGGGAGGTG
```

C---AAF ↔ CAAAAAF

FG--C' ↔ FKKGC′

Figure S5 – continued

```
                           A                                        B
D18_HSA  CCTCTGCCCGGCCGCCACCCCGTCTGGGAAGTGAGGAGCG-TCTCTGCCTGGCTGCCCATCGTCTGGGATGTGAGGAGCC-
D18_PTR  CCTCTGCCCGGCCGCCACCCCGTCTGGGAAGTGAGGAGCG-TCTCTGCCTGGCTGCCCATCGTCTGGGATGTGAGGAGCC-

                           C                                        A
D18_HSA  CCTCTGCCTGGCTGCCCAGTCTGGAAAGTGAGGAGCG-TCTCTGCCCGGCCGCCATCCCATCTAGGAAATGAGGAGCG-
D18_PTR  CCTCTGCCTGGCTACCCAGTCTGGAAAGTGAGGAGCG-TCTCTGCCCGGCCGCC-------------------------

                           B′                                       C
D18_HSA  TCTCTGCCCGGCCGCCCATCGTCTGAGATGTGGGAAGCG-CCTCTGCCCCGCCGCCCCGTCTGGGATGTGATAAGCG-
D18_PTR  ----------------CATCGTCTGAGATGTGGGAAGCG-CCTCTGCCCCGCCGCCCCGTCTGGGATCTGAGGAGCG-

                           A
D18_HSA  CCTCTGCCCGGCCGCGA-------------------------------------CCCCGTCTGAGAAGTGAGGAGAC-
D18_PTR  CCTCTGCCCGGCCGCGACCCCGTCTGGGAGGTGAGGAGCG-TCTCTGCCCGGCCGCCCCGTCTGAGAAGTGAGGAGAC-
                           A                                        C
                           A                                        A
D18_HSA  CCTCTGCCTGGCAACCGCCCCGTCTGAGAAGTGAGGAGTC-CCTCCGCCCGGCAGCCACCCCGTCTGAGAAGTGAGGAGCC-
D18_PTR  CCTCTGCCTGGCAACCACCCTGTCTGAGAAGTGAGGAGTC-CCTCCGCCCGGCAGCCGCCCCGTCTGAGAAGTGAGGAGCC-

                           A                                                F
D18_HSA  CCTCCCTCCGTCAGCCACCCCGTCTGGGAAGTGAGGAGCG-TCTCCACCCGGCAGCCACCCCGTCCGGGAGGGAGGTGGGGGGGTCAGC-
D18_PTR  CCTCCCTCCGTCAGCCACCCCGTCTGGGAAGTGAGGAGCG-TCTCCGCCCAGCAGCCACCCCGTCCGGGAGGGAGGTGGGGGGGTCAGC-

                           G                                        C′
D18_HSA  CCTCCGCCCGGCCAGCCGCCCCGTCCGGGAGGTGCGGGGCG-CCTCTGCCCAACCGCCCCTACTGGGAAGTGAGGAGCC-
D18_PTR  CCCCCGCCCGGCCAGCC--------------------------------------------------------------

                           K                                                G
D18_HSA  CCTCTGCCCGGCCAGCTGCCCCGTCCGGGAGGGAGGTGGGGGGGGTCAGC-CCCCCGCCCGGCCAGCCGCCCCGTCCGGGAGGTGAGGGGTG-
D18_PTR  -----------------GCCCCGTCCGGGAGGGAGGTGGGGGGGGTCAGC-CCCCCGCCCGGCCAGCCGCCCCATCCGGGAGGTGAGGGGCG-

                           C′                                 T
D18_HSA  CCTCTGCCCGGCCGCCTCTACTGGGAAGTGAGGAGCC-CCTCTGCCCGGCCACCACCCTGTCTGGGAGGTC
D18_PTR  CCTCTGCCCGGCCGCCCCTACTGGGAAGTGAGGAGCC-CCTCTGCCCGGCCACCACCCGGTCTGGGAGGTG
```

CAB′ ↔ C-B′

CA-A ↔ CACA

FGC′KG ↔ F--KG

Figure S5 – continued

```
                          A                                        B
D19_HSA  CCTCTGCCCGGCCGCCACCCCGTCTGGGAAGTGAGGAGCG-TCTCTGCCCGGCCGCCCATCGTCTGGGATGTGAGGAGCC-
D19_PTR  CCTCTGCCCGGCCGCCACCCCGTCTGGGAAGTGAGGAGCG-TCTCTGCCCGGCCGCCCATCGTCTGGAATGTGAGGAGCC-

                          C                                        A
D19_HSA  CCTCTGCCTGGCTGCCTAGTCTGGAAAGTGAGGAGGG-TCTCTGCCCGGCCGCCATCCCATCTAGGAAGTGAGGAGCG-
D19_PTR  CCTCTGCCTGGCTCCCTAGTCTGGAAAGTGAGGAGCG-TCTCTGCCCGGCCACCATCCCATCTAGGAAGTGAGGAGCG-

                          A                                        B′
D19_HSA  CCTCTTCCCGGCCGCCATCCCATCTGGGAAGTGAGGAGCG-TCTCTGCCCGGCCACCCATCGTCTGAGATGTGGGGAGCA-
D19_PTR  CCTCTTCCCGGCCGCCATCCCATCTGGGAAGTGAGGAGCG-TCTCTGCCCGGCCACCCATCGTCTGAGATGTGGGGAGCA-

                          C                                        C
D19_HSA  CCTCTGCCCTGCCGCCCCGTCCGGGATGTGAGGAGCG-TCTCTGCCCGGCCGCCCCGTCTGAGAAGTGAGGAGAC-
D19_PTR  CCTCTGCCCTGCCGCCCCGTCCGGGATGTGAGGAGCA-TCTCTGCCCAGCCACCCCGTCTGAGAAGTGAGGAGAC-

                          A                                        A
D19_HSA  CCTCTGCCTGGCAACCGCCCCGTCTGAGAAGTGAGGAGCC-CCTCCGCCCGGCAGCCGCCCGGTCTGAGAAGTGAGGAGCC-
D19_PTR  CCTCTGCCT---AACCGCCCCGTCTGAGAAGTGAGGAGCC-----------------------------------------
                          C
                          A                                               F
D19_HSA  CCTCCGCCCAGCAGCCACCCCGTCTGGGAAGTGAGGAGCG-TCTCTGCCCGGCAGCCGCCCCGTCCGGGCGGGAGGTGGGGGG-TCAGC-
D19_PTR  CCTCCGCCCAGCAGCCACCCCGTCTGGGAAGTGAGGAGCG-TCTCTGCCCGGCAGCCACCTCGTCCGGGAGGGAGGTGGGGGGGTCAGC-

                          G                                        C′
D19_HSA  CCCCCGCCCGGCCAGCCGCCCCGTCCGGGAGGTGAGGGGCG-CCTCTGCCCCGCCGCCCCTACTGGGAAGTAAGGAGCC-
D19_PTR  CCCCCGCCCGGCCAGCCGCCCCGTCCGGGAGGTGAGGGGTG-CCTCTGCCCGGCCACCCCTACTGGGAAGTGAGGAGCC-

                          T
D19_HSA  CCTCTGCCCGGCCACCACCCCGTCTGGGAGGTG
D19_PTR  CCTCTGCCCGGCCACCACCCCGTCTGGGAGGTG
```

CAAA ↔ C-CA

```
                          A                                        B
D20_HSA  CCTCTGCCCGGCTGCCACCCCATCTGGGAAGTGAGGAGCG-TCTCTGCCTGGCCGCCCATCGTCTGGGATGTGAGGAGCC-
D20_PTR  CCTCTGCCCGGTTGCCACCCTGTCTGGGAAGTGAGGAACG-TCTCTGCCTGGCCACCCATCATCTGGGATGTGAGGAGCC-

                          C                                        A
D20_HSA  CCTCTGCCTGGCTGCCCAGTCTGGGAAGTAAGGAGCG-CCTCTTCCCCGCCGCCATCCCATCTAGGAAGTGAGGAGCG-
D20_PTR  CCTCTGCCTGGCTGCCCAGTCTGGGAAGTAAGGAGCG-CCTCTTCCCCGCTGCCATCCCATCTAGGAAGTGAGGAGCG-

                          B′                                       C
D20_HSA  TCTCTGCCCGGCCGCCCATCCTCTGAGATGTGGGGAGCA-CCTCTGCCCCGCCGCCCCTTCTGGGATATGAGGAGCG-
D20_PTR  TCTCTGCCCGGCTGCCCATCCTCTGAGATGTGGGGAGCA-CCTCTGCCCCACCGCCCCGTCTGGGATATGAGGAGCG-

                          C                                        A
D20_HSA  TCTCTGCCTGGCCGCCCCGTCTGAGAGGTGAGGAGCC-CCTCCGCCCGGCAGCCGCCCCGTCTGAGAAGTGAGGAGGC-
D20_PTR  TCTCTGCCTGGCCACCCGGTCTGAGAGGTGAGGAGCC-CCTCCGCCCGGCAGCCGTCCTGTCTGAGAAGTGAGGAGCC-

                          F                                                      K
D20_HSA  CCTCCGCCCGGCAGCCGCCCCGGCCGGGAGGGAGGTGG-GGAGGTCAG-CCCCGCCCGGCCAGCCACCCCGTCGGGAGGGAGGTGGGGTGGGGGGGGGGTCAG-
D20_PTR  CCTCTGCCCGGCAGCCGCCCCGGTCGGGAGGGAGATGGTGGGGGTCAG-CCCCCGCCTGGCCAGCCGCCTGGTCGGGAGGGAGGTGGGG------GGGGTCAG-

                          ?
D20_HSA  CCCCCGCCCGGCCAGCCGCCCCGCCCGGTAGGGAGGGGGGGGGTCAG-------------------------------------------
D20_PTR  CCCCCGCCTGGCCAGCCGCCCCGCCCGGGAGGGAGGTGGGG-GTC-G-CCTCCGCCCGGCCAGCCGCTCCGTCCGGGAGGTGGGGGGCG-
                          ?                                               G
                          C′                                 T
D20_HSA  CCCCTGCCCAGCCGCCCCTTCTGGGAAGTGAGGAGCC-CCTCTGCCCGGCCACCACCCCGTCTGGGAGGTG
D20_PTR  CCTCTGCCCGGCCGCCCCTTCTGGGAAGTGAGGAGCC-CCTCTGCCCGGCCACCACCCCGTCTGGGAGGTG
```

K?-C′ ↔ K?GC′

Figure S6 Alignments of the VNTR regions of *Nomascus leucogenys* LAVA elements that have amplified as parts of segmental duplications. Microhomologies belonging to the same breakpoints are highlighted in identical color. Repeat unit (RU) types are annotated on top of the sequences. Position information for the elements is provided in Additional file 3.

```
                       A                                        C
1443  CCCCTGCCTGGCCGCCGCCCCATCTGGGAAGTGAGGAGCG-CCCCTGCCCGGCCGCCCCGTCTGGGAGGTGAGGAGCG--
1458  CCCCTGCCTGGCCGCCGCCCCATCTGGGAAGTGAGGAGCG-CCCCTGCCCGGCCGCCCCGTCTGGGAGGTGAGGAGCG--
1258  CCCCTGCCCGGCCGCCACCCCA--------------------------------------TCTGGGAAGTGAGGAGCG--
1370  CCTCTGCCCGGCCGCCGCCCCA--------------------------------------TCTGGGAAGTGAGGAGCG--
1371  CCCCTGCCCGGCCGCCGCCCCA--------------------------------------TGTGGGAAGTGAGGAGCG--
1341  CCCCTGCCCCGCCGCCGCCCCA--------------------------------------TCTGGGAAGTGAGGAGCG--
1342  CCCCTGCCCGGCCGCCGCCCCATCTGGGAAGTGAGGAGCG-CCCCTGCCCGGCCGCCCCGTCTGGGAGATGAGGAGCG--
1377  CCCCTGCCTGGCCGCCGCCCCATCTGGGAAGTGAGGAGGG-CCCCTGCCCGGCCGCCCCGTCTGGGAGGTGAGGAGCG--

                       C                                        C
1443  TCTCTGCCCGGCTGCCCAGTCTGGGAAGTGAGGAGCG-TCTCTGCCTGGCCACCCCGTCTGGGAAGCGAGGAGCG-----
1458  TCTCTGCCCTGCTGCCCAGTCTGGGAAGTGAGGAGCG-TCTCTGCCAGGCCACCCCGTCTGGGAAGCGAGGAGCG-----
1258  TCTCT---------------------------------------------------------------------------
1370  TCTCT---------------------------------------------------------------------------
1371  TCTCT---------------------------------------------------------------------------
1341  TCTCTGCC--------------------------------------CGGCCGCCCCGTCTGGGAAGTGAGGAGCG-----
1342  TCTCTGCC--------------------------------------TGGCCACCCCGTCTGGGAAGCGAGGAGCG-----
1377  TCTCTGCC--------------------------------------TGGCCACCCCGTCTGGGAAGCGAGGAGCG-----

                       C                                    T
1443  GCCCTGCCCGGCCGCCCCGTCTGGGATGTGGTGAGTG-CCTCTGCCCGAACGCCCCGTCTGGGAGGT
1458  GCCCTGCCTGGCCGCCCCGTCTGGGATGTGGTGAGTG-CCTCTGCCCGAACGCCCCGTCTGGGAGGT
1258  -----GCCCGGCCGCCCCATCTGGGCTGTGGTGAGTG-CCTCTGCCCGGCTGCCCCGTCTGGGAGGT
1370  -----GCCCGGCCGCCCCGTCTGGGATGTGGTGAGTG-CCTCTGCCTGGCCGCCCCATCTGGGAGGT
1371  -----GCCCGGCCGCCCCGTCTGGGATGTGGTGAGTG-CCTCTGCCCGGCCGCCCCGTCTGGGAGGT
1341  CCCCTGCCCGGCCGCCCCGTCTGGGATGTGGTGAGTG-CCTCTGCCCGGCCGCCCCGTCTGGGAGGT
1342  GCC--------------------------------------CTGCCCGGCCGCCCCGTCTGGGAGGT
1377  GCC--------------------------------------CTGCCCGGCCGCCCCGTCTGGGAGGT
```

Figure S7 A Alignment of the "inactive" LAVA_F element amplified from *Nomascus leucogenys* genomic DNA to the reference sequence (GGSC Nleu3.0/nomLeu3; chr9:35,010,156-35,012,794). Target Site Duplications are highlighted in red.

```
Reference  TTCCTCCTTTACCTCTTTTCACCCTTTAGAAATATTTTCAGTGTAAAAATGCTTCCATGAGCTAGAAGCTGTGCTCACTGTTTTGCAAAGAAAAGAGAAAGAGACCTTCCTTCCTTCCTTCCTTCCTTCCTTCTTTCTTTCTCTTTCCCT
Amplified  TTCCTCCTTTACCTCTTTTCACCCTTTAGAAATATTTTCAGTGTAAAAATGCTTCCATGAGCTAGAAGCTGTGCTCACTGTTTTGCAAAGAAAAGAGAAAGAGACCTACCTTCCTTCCTTCCTTCCTTCCTTCTTTCTTTCT--------

                                                                   ----------------------------------------------------------------------------------------------
Reference  CCTTCCTTCCTTCCTTCCTTCCTTCCTTCTTTCTTTCTTTCTTTCTCTTTCCCTCTGTGCCCCAGGCTGCAATCTACTCAGCTCGCTGCTGCCCGCGCCTCGGACTGCCTGGGACTGCCGCCACACGCCACCGCTGCCTGCTTTTTCTCG
Amplified  ------------------------------------------TTCTCTTTCCCTCTGTGCCCCAGGCTGCAATCTACTCAGCTCGCTGCTGCCCGCGCCTCGGACTGCCTGGGACTGCCGCCACACGCCACCGCTGCCTGCTTTTTCTCG
                                                Alu-like
           ------------------------------------------------------------------------------------->
Reference  TTTGGCTGCAGGCGCGCGGTCGCCATGTTGGCCACGCTGCTCGCCAGCTCCTGACGCCGAGTGCTCTGCCCGCCTCAGCCTCCCGAGCCCCTGCCCGGCCACCGCCCCGTCTGGGACGTGGGGAGCGCCTCTGCCCGGCCGCCCCGTCCG
Amplified  TTTGGCTGCAGGCGCGCGGTCGCCATGTTGGCCACGCTGCTCGCCAGCTCCTGACGCCAAGTGCTCTGCCCGCCTCAGCCTCCCGAGCCCCTGCCCGGCCACCGCCCCGTCTGGGACGTGGGGAGCGCCTCTGCCCGGCCGCCCCGTCCG

Reference  GGAAGAAGTGAGGAGCGCCTCTGCCCGGCCGCCCCGTCTGGGAAGTGAGGAGCGCCTCTGCACGGCCACCCATCGTCTGGGAAGTGAGGAGTGCCTCTGCCCGGCCGCCCCGTCTGGGAAGTGAGGAGCGCCTCTGCCCGGCCACCCACC
Amplified  GGAAGAAGTGAGGAGCGCCTCTGCCCGGCCGCCCCGTCTGGGAAGTGAGGAGCGCCTCTGCACGGCCACCCATCGTCTGGGAAGTGAGGAGCGCCTCTGCCCGGCCGCCCCGTCTGGGAAGTGAGGAGCGCCTCTGCCCGGCCACCCACC

Reference  GTCTGGGAAGTGAGGAGCACCTCTGCCCGGCCACCCACCGTCTGGGAAGTGAGGAGCGCCTCTGCCCAGCCGCCCCGTCAGGGAAGTGAGGAGCGCCTCTGCCCGGCCGCCCCATCTGTGAAATGAGGAGCGCCTCTGCCCGGCCACCTA
Amplified  GTCTGGGAAGTGAGGAGCACCTCTGCCCGGCCACCCACCGTCTGGGAAGTGAGGAGCGCCTCTGCCCAGCCGCCCCGTCAGGGAAGTGAGGAGCGCCTCTGCCCGGCCGCCCCATCTGTGAAATGAGGAGCGCCTCTGCCCGGCCACCTA

Reference  TCGTCTGGGAGGTGAGGAGCGCCTCTGCCCGGCCGCCCCGTCCGGGAAGAAGTGAGGAGCGCCTCTGCCCGGCCGCCCGTCCGGGAAGAAGTGAGGAGCGCCTCTGCCCGGCTGCCCGTATGGGAGGTGAGGAGAACCTCTGCCCGGCCA
Amplified  TCGTCTGGGAGGTGAGGAGCGCCTCTGCCCGGCCGCCCCGTCCGGGAAGAAGTGAGGAGCGCCTCTGCCCGGC-----------------------------------------------------------------------------

Reference  CCCATCGTCTGGGAGGTGAGGAGCGCCTCTGCCCGGCCACCCACCGTCTGGGAAGTGAGGAGCGCCTCTGCCCGGCCGCCCCGTCTGGGAAGTGAGGAGCGCCTCTGCCCGGCCGTCCCGTCTGGGAAGTGAGGAGCGCCTCTGCCCGGC
Amplified  ------------------------------------------------------------------------------------------------------------------------------------------------------

Reference  CACCCACCGTCTGGGAAGTGAGGAGCGCCTCTGCCCGGCCACCCACCGTCTGGGAAGTGAGGAGCGCCTCTGCCCGGCCACCCACCGTCTGGGAAGTGAGGAGCGCCTCTGCCCGGCCACCCACCGTCTGGGGAAGTGAGGAGCGCCTCT
Amplified  ------------------------------------------------------------------------------------------------------------------------------------------------------

Reference  GCCCGGCCACCCACCATCTGGGAAGTGAGGAGCGCCTCTGCCCGGCCACCCCGTCTGGGAAGTGAGGAGCGCCTCTGCCCGGCCGCCCCATCTGTGAAATGAGGAGCGCCTCTGCCCAGCCACCTATCGTCTGGGAGGTGAGGAGCGCCT
Amplified  ------------------------------------------------------------------------------------------------------------------------------------------------------

Reference  CTGCCCGGCCGCCCCGTCCGGGAAGAAGTGAGGAGCACCTCTGCCCGGCCGCCCCATCCGGGAAGAAGTGAGGAGCGCCTCTGCCCGGCCGCCCCGTCCGGGAAGAAGTGAGGAGCGCCTCTGCCCGGCCGCCCCATCCGGGAAGAAGTG
Amplified  -------------------------------------------------TGCCCCGTCCGGGAAGAAGTGAGGAGCGCCTCTGCCCGGCCGCCCCGTCCGGGAAGAAGTGAGGAGCGCCTCTGCCCGGCCGCCCCATCTGGG---AAGTG

Reference  AGGAGCGCCTCTGCCCGGCCGCCCCGTCCGGGAAGAAGTGAGGAGCGCCTCTGCCCGGCCGCCCCGTCCGGGAAGAAGTGAGGAGCGCCTCTGCCCTGCCGCCCCGTCTGGGAAGTGAGGAGCGCCTCTGCCCGGCCGCCCCGTCCGGGA
Amplified  AGGACCGCCTCTGCCCGGCCGCCCCGTCCGGGAAGAAGTGAGGAGCGCCTCTGCCCGGCCGCCCCGTCCGGGAAGAAGTGAGGAGCGCCTCTGCCCGGCCGCCCCGTCTGGGAAGTGAGGAGCGCCTCTGCCCGGCCGCCCCGTCCGGGA

Reference  AGAAGTGAGGAGCGCCTCTGCCCGGCCGCCCTGTCTGGGAAGTGAGGAGCGCCTCGGCCCGGCCGCCCCGTCTGGGAAGTGAGGAGCGCCTCTGCCCGGCCGCCCCGTCTGGGAAGTGAGGAGCGCCTCTGCCTGGCCGCCCCGTCTGGG
Amplified  AGAAGTGAGGAGCGCCTCGGCCCGGCCGCCCTGTCTGGGAAGTGAGGAGCGCCTCGGCCCGGCCGCCCCGTCTGGCAAGTGAGGAGCGCCTCTGCCCGGCCGCCCCGTCTGGGAAGTGAGGAGCGCCTCTGCCCGGCCGCCCCGTCTGGG

Reference  AAGTGAGGAGCACCTCTGCCCGGCCACCCATCGTCTGGGAAGTGAGGAGCGCCTCTGCCCGGCCACCCATTGTCTGGGAAGTGAGGGGCGCCTCTGCCCGGCCACCTATCGTCTGGGAAGAAGTGAGGAGCGTCTCTGCCTGGCCGCCCC
Amplified  AAGTGAGGAGCACCTCTGCCCGGCCACCCATCGTCTGGGAAGTGAGGAGCGCCTCTGCCCGGCCACCCATCGTCTGGGAAGTGAGGGGCGCCTCTGCCCGGCCACCTATCGTCTGGGAAGAAGTGAGGAGCGTCTCTGCCT---------

                                                                                                     ------------------------------------------------------------
Reference  GTCTGGGAAGTGAGGAGCGCCTCTGCCCGGCCGCCCCGTGTCTGGGAAGAAGTGAGGAGCGCCTCTGCCCGGCCGCTCCGTCTGGGAGGTCTACAATGGAGGCCAGAAGCAATGTGGGGGCTGGTCGTGGTGGCTCACGCCTGTGGTCCC
Amplified  ----------------------------GGCCGCCCCGTGTCTGGGAAGAAGTGAGGAGCGCCTCTGCCCGGCCGCTCCGTCTGGGAGGTCTACAATGGAGGCCAGAAGCAATGTGGGGGCTGGTCGTGGTGGCTCACGCCTGTGGTCCC

           ------------------------------------------------------------------------------------------------------------------------------------------------------
Reference  AGCACTCTGGGGGGCGAGGCGGGTTGATCACTTCGGACTAGGAGTTCGAGACCAGTCTGGCCAACTTGGCGAAACATGAAAAATACAACAGACAAACCAACCAACCAACTCAGAGACAACAAAACAGGTCTACCCTGGAGTCATACTCTA
Amplified  AGCACTCTGGGGGGCGAGGCGGGTTGATCACTTCGGACTAGGAGTTCGAGACCAGTCTGGCCAACTTGGCGAAACATGAAAAATACAACAGACAAACCAACCAACCAACTCAGAGACAACAAAACAGGTCTACCCTGGAGTCATACTCTA
```

3’ part

```
           --------------------------------------------------------------------------------------------------------------------------------------------------------------------------------------------------------------------------------
Reference  ATTTTTTCTATTTTCCTCCCTTTCTGATCCTTTATCCCACTTTCTTTTTCTTCCTCTTCCTTCTCCCTCTTCTTTGTGAAATAGAGGATTGAGTTATTATCACTGATCCACATAAAGTACCTCTCTACCTTATTTTAACTCCCACCCCCC
Amplified  ATTTTTTCTATTTTCCTCCCTTTCTGATCCTTTATCCCACTTTCTTTTTCTTCCTCTTCCTTCTCCCTCTTCTTTGTGAAATAGAGGATTGAGTTATTATCACTGATCCACATAAAGTACCTCTCTACCTTATTTTAACTCCCACCCCCC

           --------------------------------------------------------------------------------------------------------------->
Reference  ATTTCTATTCCCCGACTTCCCATGTGTAACCTTCCTAATATGTTTGATACGCATCTTTTTGTTTGTATGTATTTTTAGAAAATGTTTATTGTTTTTGTGTGCAAAAAAAAAAAATTAATAAAAAAAAAAGAGAAAGAGAAAAAGGAAAAGA
Amplified  ATTTCTATTCCCCGACTTCCCATGTGTAACCTTCCTAATATGTTTGATACGCATCTTTTTGTTTGTATGTATTTTTAGAAAATGTTTATTGTTTTTGTGTGCAAAAAAAAAAAATTAATA-AAAAAAAAGAGAAAGAGAAAAAGGAAAAGA

Reference  AAAAAATATAGTTTAATAACATATAAACACCATAGTATATCTGAATTTATTAATACTCCCACTACAGATGGCAGTAAGCACTACAGAGC
Amplified  AAAAAATATAGTTTAATAACATATAAACACCATAGTATATCTGAATTTATTAATACTCCCACTACAGATGGCAGTAAGCACTACAGAGC
```

Figure S7 B Fine structure of the domain junctions in LAVA chimeras

L_EL_F 5’: BstAPI-blunted/Ava-blunted

```
LAVA_E CGCCTGCCTTGGCCTACCAGGGTGCTGGGATTGCAG cccctgcccggccgccgccccgtctgggaggtgggg
LELF 5’CGCCTGCCTTGGCCTACCAGGGTGCTGGGATTGCAGGcccctgcccggccaccgccccgtctgggaggtgggg
LAVA_F TGAGGCCGAGTGCTCTGCCCGCCTCAGCCTCCCGA Gcccctgcccggccaccgccccgtctgggaggtgggg
```

L_FL_E 5’: SmaI/BstAPI-blunted

```
LAVA_F TGAGGCCGAGTGCTCTGCCCGCCTCAGCCTCCCGAGcccctgcccggccaccgccccgtctgggaggtgggg
LFLE 5’TGAGGCCGAGTGCTCTGCCCGCCTCAGCCTCCCGAGcccctgcccggccgccgccccgtctgggaggtgggg
LAVA_E CGCCTGCCTTGGCCTACCAGGGTGCTGGGATTGCAGcccctgcccggccgccgccccgtctgggaggtgggg
```

L_EL_F 3’: AccI-blunted/RsaI

```
LAVA_E aggagcgcttctgcccggccgctccgtctgggaggtCTACCACCGAGGCCAGAAGCAATGTGGGGGCTGGAC
LELF 3’aggagcgcttctgcccggccgctccgtctgggaggtCTACCATGGAGGCCAGAAGCAATGTAGGGGCTGGAC
LAVA_F aggagcgcctctgcccggccgctccgtctgggaggtCTACCATGGAGGCCAGAAGCAATGTAGGGGCTGGAC
```

L_FL_E 3’: AccI-blunted/RsaI

```
LAVA_F aggagcgcctctgcccggccgctccgtctgggaggtCTACCATGGAGGCCAGAAGCAATGTAGGGGCTGGAC
LFLE 3’aggagcgcctctgcccggccgctccgtctgggaggtCTACCATGGAGGCCAGAAGCAATGTAGGGGCTGGAC
LAVA_E aggagcgcttctgcccggccgctccgtctgggaggtCTACCACCGAGGCCAGAAGCAATGTGGGGGCTGGAC
```

$L_EL_FL_E$: 5’ end - BstAPI-blunted/Ava-blunted; 3’ end - AccI-blunted/RsaI

```
LAVA_E CGCCTGCCTTGGCCTACCAGGGTGCTGGGATTGCAG cccctgcccggccgccgccccgtctgggaggtgggg
LELF 5’CGCCTGCCTTGGCCTACCAGGGTGCTGGGATTGCAGGcccctgcccggccaccgccccgtctgggaggtgggg
LAVA_F TGAGGCCGAGTGCTCTGCCCGCCTCAGCCTCCCGA Gcccctgcccggccaccgccccgtctgggaggtgggg
LAVA_F aggagcgcctctgcccggccgctccgtctgggaggtCTACCATGGAGGCCAGAAGCAATGTAGGGGCTGGAC
LFLE 3’aggagcgcctctgcccggccgctccgtctgggaggtCTACCATGGAGGCCAGAAGCAATGTAGGGGCTGGAC
LAVA_E aggagcgcttctgcccggccgctccgtctgggaggtCTACCACCGAGGCCAGAAGCAATGTGGGGGCTGGAC
```

$L_FL_EL_F$: 5’ end - SmaI/BstAPI-blunted; 3’ end - AccI-blunted/RsaI

```
LAVA_F TGAGGCCGAGTGCTCTGCCCGCCTCAGCCTCCCGAGcccctgcccggccaccgccccgtctgggaggtgggg
LFLE 5’TGAGGCCGAGTGCTCTGCCCGCCTCAGCCTCCCGAGcccctgcccggccgccgccccgtctgggaggtgggg
LAVA_E CGCCTGCCTTGGCCTACCAGGGTGCTGGGATTGCAGcccctgcccggccgccgccccgtctgggaggtgggg
LAVA_E aggagcgcttctgcccggccgctccgtctgggaggtCTACCACCGAGGCCAGAAGCAATGTGGGGGCTGGAC
LELF 3’aggagcgcttctgcccggccgctccgtctgggaggtCTACCATGGAGGCCAGAAGCAATGTAGGGGCTGGAC
LAVA_F aggagcgcctctgcccggccgctccgtctgggaggtCTACCATGGAGGCCAGAAGCAATGTAGGGGCTGGAC
```

A_I_I: exchange at the 5’ end using common AvaI site

```
Active TGAGGCCGAGTGCTCTGCCCGCCTCAGCCTCCCGAGcccctgcccggccaccgccccgtctgggaggtgggg
A_I_I  TGAGGCCGAGTGCTCTGCCCGCCTCAGCCTCCCGAGcccctgcccggccaccgccccgtctgggacgtgggg
Inact. TGACGCCAAGTGCTCTGCCCGCCTCAGCCTCCCGAGcccctgcccggccaccgccccgtctgggacgtgggg
```

I_A_A: exchange at the 5’ end using common AvaI site

```
Inact. TGACGCCAAGTGCTCTGCCCGCCTCAGCCTCCCGAGcccctgcccggccaccgccccgtctgggacgtgggg
I_A_A  TGACGCCAAGTGCTCTGCCCGCCTCAGCCTCCCGAGcccctgcccggccaccgccccgtctgggaggtgggg
Active TGAGGCCGAGTGCTCTGCCCGCCTCAGCCTCCCGAGcccctgcccggccaccgccccgtctgggaggtgggg
```

A_A_I: exchange at the VNTR 3’ end using the common MbiI site

```
Active aggagcgcctctgcccggccgctccgtctgggaggtCTACCATGGAGGCCAGAAGCAATGTAGGGGCTGGAC
A_A_I  aggagcgcctctgcccggccgctccgtctgggaggtCTACAATGGAGGCCAGAAGCAATGTGGGGGCTGGTC
Inact. aggagcgcctctgcccggccgctccgtctgggaggtCTACAATGGAGGCCAGAAGCAATGTGGGGGCTGGTC
```

I_I_A: AccI-blunted/RsaI

```
Inact. aggagcgcctctgcccggccgctccgtctgggaggtCTACAATGGAGGCCAGAAGCAATGTGGGGGCTGGTC
I_I_A  aggagcgcctctgcccggccgctccgtctgggaggtCTACCATGGAGGCCAGAAGCAATGTAGGGGCTGGAC
Active aggagcgcctctgcccggccgctccgtctgggaggtCTACCATGGAGGCCAGAAGCAATGTAGGGGCTGGAC
```

A_I_A: 5’ end – AvaI; 3’ end - AccI-blunted/RsaI

```
Active TGAGGCCGAGTGCTCTGCCCGCCTCAGCCTCCCGAGcccctgcccggccaccgccccgtctgggaggtgggg
A_I_I  TGAGGCCGAGTGCTCTGCCCGCCTCAGCCTCCCGAGcccctgcccggccaccgccccgtctgggacgtgggg
Inact. TGACGCCAAGTGCTCTGCCCGCCTCAGCCTCCCGAGcccctgcccggccaccgccccgtctgggacgtgggg
Inact. aggagcgcctctgcccggccgctccgtctgggaggtCTACAATGGAGGCCAGAAGCAATGTGGGGGCTGGTC
I_I_A  aggagcgcctctgcccggccgctccgtctgggaggtCTACCATGGAGGCCAGAAGCAATGTAGGGGCTGGAC
Active aggagcgcctctgcccggccgctccgtctgggaggtCTACCATGGAGGCCAGAAGCAATGTAGGGGCTGGAC
```

I_A_I: 5’ end – AvaI; 3’ end - MbiI

```
Inact. TGACGCCAAGTGCTCTGCCCGCCTCAGCCTCCCGAGcccctgcccggccaccgccccgtctgggacgtgggg
I_A_A  TGACGCCAAGTGCTCTGCCCGCCTCAGCCTCCCGAGcccctgcccggccaccgccccgtctgggaggtgggg
Active TGAGGCCGAGTGCTCTGCCCGCCTCAGCCTCCCGAGcccctgcccggccaccgccccgtctgggaggtgggg
Active aggagcgcctctgcccggccgctccgtctgggaggtCTACCATGGAGGCCAGAAGCAATGTAGGGGCTGGAC
A_A_I  aggagcgcctctgcccggccgctccgtctgggaggtCTACAATGGAGGCCAGAAGCAATGTGGGGGCTGGTC
Inact. aggagcgcctctgcccggccgctccgtctgggaggtCTACAATGGAGGCCAGAAGCAATGTGGGGGCTGGTC
```

Sequences belonging to the *Alu*-like domains as well as those of the LAVA 3’ parts are given in uppercase, VNTR sequences in lowercase. Restriction endonuclease recognition sites (or parts of them - RsaI) are underlined; sequences found in the chimeras are highlighted in yellow. The additional G resulting from cloning in chimeras L_EL_F 5’ and $L_EL_FL_E$ is highlighted in black.
